# Microglia-mediated neuroinflammation in Alzheimer’s disease: mechanisms and emerging therapeutic targets

**DOI:** 10.3389/fncel.2026.1902577

**Published:** 2026-08-20

**Authors:** Lianjing Xu, Ying Zhang, Li Jiang, Jin Feng Xing, Jing Hu, Tianye Lan, Guang Ta

**Affiliations:** 1 Affiliated Hospital of Changchun University of Chinese Medicine, Changchun, China; 2 School of Integrated Traditional Chinese and Western Medicine, Changchun University of Chinese Medicine, Changchun, China; 3 School of Traditional Chinese Medicine, Changchun University of Chinese Medicine, Changchun, China

**Keywords:** Alzheimer’s disease, microglia, microglial cell-state heterogeneity, neuroinflammation, pathogenesis, therapeutic targets

## Abstract

Alzheimer’s disease is a complex neurodegenerative disorder characterized pathologically by amyloid-β deposition and pathological tau aggregation. Amyloid-β deposition typically occurs during the preclinical stage; however, amyloid burden does not exhibit a simple linear relationship with neurodegeneration or cognitive decline. In contrast, the spatial distribution of tau pathology is more closely associated with clinical progression. As the resident innate immune cells of the central nervous system, microglia participate in the recognition, uptake, and containment of amyloid-β and tau. Nevertheless, persistent exposure to damage-associated signals can lead to lysosomal dysfunction, dysregulated lipid metabolism, and mitochondrial impairment in microglia, thereby amplifying neuroinflammation, aberrant synaptic elimination, and neuronal injury. The traditional binary M1/M2 classification is inadequate to capture the continuous, overlapping, and context-dependent functional states of microglia, which vary across brain regions, genetic backgrounds, and disease stages. This review integrates recent evidence from genetic, single-cell/single-nucleus, and spatial transcriptomic studies and proposes a “cellular state–pathological network–therapeutic window” framework. We systematically discuss the roles of microglia in amyloid-β plaque seeding and compaction, NLRP3 inflammasome activation, mitochondrial DNA–cGAS–STING signaling, complement-mediated synaptic engulfment, and bidirectional microglia–tau feedback. On this basis, we critically evaluate the mechanistic rationale, stage dependence, and translational limitations of therapeutic axes involving TREM2/CD33, P2X7–NLRP3 and cGAS–STING, CSF1R/complement, and TNF–TNFR1–RIPK1. Current evidence suggests that the key to microglia-targeted therapy is not the broad activation or suppression of immune responses, but rather the biomarker-guided and disease-stage-specific modulation of pathogenic signaling while preserving homeostatic functions such as plaque containment, debris clearance, synaptic maintenance, and tissue repair.

## 1 Introduction

Alzheimer’s disease (AD) is the most common type of dementia among the elderly. It is a neurodegenerative disease of the central nervous system, characterized by gradual cognitive deterioration as well as non-cognitive neuropsychiatric symptoms (Ballard et al., 2011). As population aging becomes a worldwide trend, AD has become a major public health issue. Its high incidence and disability rates place a substantial economic and caregiving burden on society and families. Current symptom-oriented pharmacological treatments for Alzheimer’s disease (AD) primarily include acetylcholinesterase inhibitors, such as donepezil, rivastigmine, and galantamine, as well as the N-methyl-D-aspartate receptor antagonist memantine. These agents provide modest symptomatic benefits but do not halt the underlying neurodegenerative process (Geldmacher, 2024). In recent years, amyloid-targeting monoclonal antibodies, including lecanemab and donanemab, have modestly slowed cognitive and functional decline in selected patients with biomarker-confirmed early AD (Sims et al., 2023; van Dyck et al., 2023). However, no currently available therapy can halt or reverse the progression of AD, underscoring the need for the continued development of novel therapeutic strategies.

From the viewpoint of genetics, AD can be clearly classified into familial AD (FAD) and sporadic AD (SAD). The former largely results from genetic mutations affecting amyloid precursor protein (APP) or APP-processing enzymes, particularly γ-secretase and its presenilin components, PS1 and PS2. The latter is closely associated with age, genetic risk factors (such as APOEε4), and environmental factors. Regardless of type, AD is pathologically characterized by excessive deposition of β-amyloid (Aβ) and the formation of neurofibrillary tangles (NFTs) caused by hyperphosphorylated tau aggregation (Selkoe, 2011). These changes trigger a cascade of neuroinflammation and oxidative stress, and are also key factors in neuronal dysfunction and death. Because neurodegenerative diseases are chronic and progressive, the core pathogenesis of AD is not fully understood. The amyloid cascade hypothesis and tau hypothesis remain the primary recognized mechanisms underlying AD’s pathological progression (Galimberti and Scarpini, 2012). The amyloid cascade hypothesis proposes that an imbalance between Aβ production and clearance, followed by the accumulation of aggregation-prone Aβ species, represents an early event that triggers downstream tau dysregulation, synaptic dysfunction, glial responses, and neurodegeneration. In contrast, tau-centered models emphasize that abnormal phosphorylation, misfolding, aggregation, seeding, and propagation of tau disrupt microtubule stability, axonal transport, and neuronal function. Tau burden and its spatial distribution are more closely associated with regional neurodegeneration and clinical impairment than amyloid burden. Therefore, these hypotheses should be viewed as complementary rather than mutually exclusive theoretical frameworks for understanding the pathogenesis of AD (Wang and Mandelkow, 2016; Long and Holtzman, 2019; La Joie et al., 2020).

However, many clinical trials aimed at clearing forms of Aβ from patients’ brains have produced disappointing results. Although patients’ amyloid burden decreased, their cognitive function did not improve (Lannfelt et al., 2014; Egan et al., 2018). Although many early Aβ-targeted clinical trials failed to demonstrate clear clinical benefits, these findings do not, in themselves, invalidate the amyloid cascade hypothesis. The negative results may reflect intervention only after substantial downstream pathology had already developed, insufficient target engagement, targeting of noncritical Aβ species, irreversible neuronal damage, or clinical and biological heterogeneity. Therefore, current evidence supports Aβ accumulation as an important early component of AD biology, while also indicating that amyloid pathology alone is insufficient to explain the full temporal, biological, and clinical heterogeneity of the disease (Karran and De Strooper, 2022; Sims et al., 2023). Several alternative hypotheses have been proposed, including oxidative stress, cholinergic dysfunction, and neuroinflammation (Hampel et al., 2018; Butterfield and Halliwell, 2019; Wang H. et al., 2019). These offer important insights into the pathogenesis of AD. Among these, neuroinflammation is critically involved in the neuropathological alterations associated with AD. Extensive research shows that neuroinflammation, mediated by sustained activation of glial cells, is a major cause of neurodegenerative lesions and cognitive impairment. Neuroinflammation is not simply a secondary outcome of AD, but rather an important factor promoting disease progression. A prominent hallmark of AD-associated neuroinflammation is the clustering of activated microglia around sites of injury (Leng and Edison, 2021). Microglia are the resident innate immune cells of the central nervous system (CNS). They participate in neurodevelopment and homeostasis by phagocytosing and clearing damaged neurons and abnormal synapses. Nevertheless, excessive activation of microglia can induce substantial secretion of pro-inflammatory cytokines and chemokines. This leads to neuronal dysfunction (Heneka et al., 2013; Hong et al., 2016). Thus, therapeutic strategies targeting microglial function represent a promising approach to intervene in AD-related neuroinflammatory responses and to delay or halt pathological progression (Leng and Edison, 2021; Sun et al., 2023). This review provides an overview of the interplay between microglia and neuroinflammatory responses in AD. We also describe established or potential therapeutic strategies targeting microglia in AD.

## 2 Microglia in AD

### 2.1 Microglial origin and developmental processes

Microglia serve as the principal innate immune cells of the CNS. They perform macrophage-like functions and make up approximately 10%–15% of glial cells. Microglia primarily arise from myeloid progenitor cells in the yolk sac during the early stages of embryonic development (Harry, 2013; Hansen et al., 2018). These progenitors become yolk sac macrophages, migrate to the CNS, and establish themselves before the blood-brain barrier matures, then differentiate into microglia (Lawson et al., 1990). Unlike peripheral macrophages, the maintenance of microglia depends predominantly on local self-renewal, rather than on replacement by bone marrow-derived monocytes (Bruttger et al., 2015).

Although microglia are present throughout the CNS, their regional distribution is heterogeneous, with higher densities in the hippocampus, basal ganglia, and substantia nigra, and lower densities in the cerebellum and brainstem (Lawson et al., 1990; Mittelbronn et al., 2001). Microglial development, differentiation, and maintenance are tightly controlled by multiple factors and depend on interactions with other cells. For instance, CX3CR1 on microglia interacts with neuron-derived C-X3-C motif chemokine ligand 1 (CX3CL1) to regulate their proliferation and activation (Hatori et al., 2002; Pawelec et al., 2020); microglial development and survival are critically dependent on CSF1R signaling and its neuron-secreted ligands, IL-34 and CSF-1 (Wang et al., 2012; Elmore et al., 2014; Easley-Neal et al., 2019). Though IL-34 and CSF-1 share a receptor, their roles differ: CSF-1 is more important during embryonic microglial development, while IL-34 is key after birth. Their regional expression also varies: IL-34 is produced mainly by gray matter neurons, crucial for gray matter microglia, whereas CSF-1 is expressed in the white matter, regulating its microglia (Easley-Neal et al., 2019). Glial cell-derived cytokines also influence microglia. Astrocyte-derived IL-33 signals via interleukin-1-like receptor 1 on microglia, promoting synapse phagocytosis during CNS development (Vainchtein et al., 2018). Activated astrocytes secrete chemokines like CCL2 and CXCL10, helping recruit microglia to injury sites (de Waard and Bugiani, 2020).

As important immune effector cells in the central nervous system (CNS), microglia are regarded as key sentinel cells that maintain homeostasis within the brain microenvironment. They express a “sensome” comprising diverse receptors that recognize exogenous pathogens and endogenous damage signals and initiate corresponding responses (Hickman et al., 2013; Prinz et al., 2019). Under physiological conditions, microglia continuously survey the brain parenchymal microenvironment through their highly dynamic processes and participate in immune surveillance, cellular debris clearance, trophic support, and tissue repair (Nimmerjahn et al., 2005; Prinz et al., 2019).

### 2.2 Morphological changes and functional-state heterogeneity of microglia

Accumulating evidence in recent years indicates that microglia contribute substantially to the development and progression of Alzheimer’s disease (AD). Owing to their pronounced phenotypic plasticity, microglia can undergo dynamic remodeling when exposed to various pathophysiological stimuli. These changes result in significant modifications in cell morphology, surface marker expression, secretory profiles, and proliferative activity. Under steady-state conditions, microglia typically exhibit a highly branched morphology. They are characterized by slender and extensively ramified processes (Boche et al., 2013). Historically, these ramified microglia were described as being in a “resting” state. Evidence from *in vivo* two-photon imaging indicates that microglial processes are remarkably dynamic, undergoing continuous extension and retraction. This motility enables microglia to monitor their microenvironment and promptly respond to potential abnormalities (Nimmerjahn et al., 2005). Transcriptomic analysis further reveals that branched-state microglia primarily express genes associated with maintaining brain homeostasis. These genes include those regulating synaptic integrity, neuronal maturation, and interactions with other glial cells involved in intracellular homeostasis (Parakalan et al., 2012). Upon detecting microenvironmental changes, microglia can rapidly migrate to injury or stimulation sites via their processes. Their movement is guided by chemotactic signals (Boche et al., 2013). Microglial activation is commonly accompanied by a morphological transformation from a highly branched morphology to an amoeboid phenotype. Activated microglia show increased cell volume, shortened processes, and increased cytoplasmic vacuoles (Madore et al., 2013). Such morphological remodeling is accompanied by functional responses, including migratory activity, antigen-presenting capacity, and phagocytic function (Parakalan et al., 2012).

However, the relationship between microglial morphology and function is not a simple one-to-one correspondence. Morphological features alone, such as a ramified or amoeboid appearance, cannot accurately define the molecular characteristics or biological functions of microglia. Previous studies have frequently used the M1/M2 polarization framework to describe functional changes in microglia. Although the M1/M2 terminology is useful for summarizing certain experimental observations, it does not adequately reflect the marked heterogeneity of microglia in vivo. Paolicelli et al. noted that binary classifications such as “resting/activated” and “M1/M2” overlook the diverse microglial states observed during development, aging, and neurological diseases, and that microglia should not be simply categorized as either beneficial or detrimental (Paolicelli et al., 2022). Within the brain’s complex microenvironment, microglia may simultaneously express genes associated with inflammation, phagocytosis, lysosomal activity, lipid metabolism, antigen presentation, interferon responses, and tissue repair. Therefore, detecting only a few markers, such as inducible nitric oxide synthase, arginase-1, CD86, or CD206, is insufficient to classify microglia as having a fixed M1 or M2 phenotype.

A more appropriate contemporary view is that microglia occupy a continuum of dynamic, overlapping, and context-dependent functional states. These states are jointly regulated by multiple factors, including age, sex, brain region, genetic background, disease stage, pathological stimuli, and intercellular interactions (Paolicelli et al., 2022). In AD, microglia may exert protective effects by phagocytosing Aβ, promoting amyloid plaque compaction, and clearing cellular debris. However, following prolonged exposure to Aβ, pathological tau, and oxidative stress, microglia may also develop lysosomal dysfunction, dysregulated lipid metabolism, sustained inflammatory signaling, and excessive complement-mediated synaptic elimination (Cai et al., 2022; McFarland and Chakrabarty, 2022).

Studies have also suggested that microglia do not merely passively accumulate around pre-existing Aβ plaques but may actively participate in the formation, propagation, and structural remodeling of Aβ pathology. Aβ-laden microglia can migrate to previously unaffected brain regions and promote local Aβ deposition (d’Errico et al., 2022). Before plaque formation, relatively homeostatic microglia may contribute to early seeding; after plaque formation, reactive microglia can promote plaque compaction and reduce surrounding neuritic damage (Baligács et al., 2024). Therefore, microglial responses may exert different or even opposing effects at different stages of the disease.

### 2.3 Transcriptomic characteristics of microglial states in AD

Advances in single-cell RNA sequencing, single-nucleus RNA sequencing, and spatial transcriptomics have further revealed the complexity of microglial states in AD. Microglial programs reported to date include homeostatic, disease-associated, inflammatory, interferon-responsive, antigen-presenting, lipid metabolism–associated, proliferative, and stress-associated states. Disease-associated microglia (DAM) are among the most extensively studied states. In animal models of AD, DAM are generally characterized by reduced expression of homeostasis-associated genes, such as *P2RY12*, *P2RY13*, *CX3CR1*, and *TMEM119*, accompanied by increased expression of genes associated with lipid metabolism, lysosomal function, and phagocytosis, including *APOE*, *TREM2*, *LPL*, *CTSB*, and *CD68* (Chen and Colonna, 2021; Singh et al., 2022). Early studies proposed that the DAM response may comprise TREM2-independent and TREM2-dependent stages. More recent evidence further indicates that TREM2 regulates microglial recognition of Aβ, clustering around plaques, plaque encapsulation, and phagocytic responses through both SYK-dependent and SYK-independent pathways (Keren-Shaul et al., 2017; Hou et al., 2022; Wang S. et al., 2022). Moreover, the response of human microglia to Aβ is not limited to a DAM-like program but encompasses multiple transcriptional states and state-specific regulation by various AD risk genes (Mancuso et al., 2024). Cross-disease transcriptomic analyses and human microglial atlases have further demonstrated that disease-associated and lipid metabolism–associated microglial programs are present in AD, multiple sclerosis, Lewy body diseases, and other neurological conditions (Guvenek et al., 2024; Martins-Ferreira et al., 2025). Therefore, DAMs are more appropriately understood as a collection of dynamic, heterogeneous, and context-dependent transcriptional programs that microglia adopt within specific pathological environments. They should not be regarded as a clearly delineated, functionally fixed, or AD-specific cellular subtype, nor should they be simply classified as either protective or detrimental (Paolicelli et al., 2022).

Prater et al. performed microglia-enriched single-nucleus RNA sequencing of the dorsolateral prefrontal cortex from 12 patients with AD and 10 controls. They identified multiple previously recognized and newly defined molecular states of microglia in the human AD brain, some of which were significantly enriched in AD samples (Prater et al., 2023). Notably, substantial subpopulation heterogeneity was evident even among microglia that retained homeostatic markers. Trajectory analysis suggested that microglia may undergo state transitions along multiple interconnected paths, rather than progressing unidirectionally from a putative “resting state” to a uniform “activated state.” Different forms of AD pathology may also elicit distinct microglial transcriptional responses. By comparing transgenic mouse models of amyloid and tau pathology, Sierksma et al. found that a microglial expression module enriched in AD risk genes responded strongly to Aβ pathology but exhibited a relatively limited response in the tau models examined (Sierksma et al., 2020). This module included the AD risk genes *APOE*, *CLU*, *INPP5D*, *CD33*, *PLCG2*, *SPI1*, and *FCER1G*, suggesting that some forms of genetic susceptibility may act by modulating microglial responses to Aβ. However, these findings do not imply that microglia are uninvolved in tau pathology; rather, they indicate that microglial transcriptional responses are jointly shaped by the type of pathology, animal model, brain region, and disease stage. Mancuso et al. transplanted human stem cell–derived microglia into the brains of mice with amyloid pathology and analyzed 138,577 single-cell transcriptomes (Mancuso et al., 2024). In addition to a DAM-like state, the human microglia displayed prominent human leukocyte antigen–associated antigen-presenting and cytokine/chemokine-responsive states. The deletion of *TREM2* or *APOE*, the *TREM2* R47H variant, and different *APOE* alleles exerted distinct effects on these states. These findings indicate that AD risk genes do not simply drive all microglia toward a single pro-inflammatory state but instead selectively regulate specific molecular and functional programs.

Overall, microglial responses in AD are dynamic, multidimensional, genetically influenced, and dependent on the pathological context. Transcriptomically defined cell clusters should not be directly equated with stable functional subtypes. Their identification may also be influenced by the brain region sampled, disease stage, postmortem interval, cell isolation method, and analytical strategy. Future studies integrating single-cell transcriptomics with spatial mapping, epigenomics, proteomics, and functional experiments will be required to accurately define the roles of distinct microglial states across different stages of AD.

**FIGURE 1:**
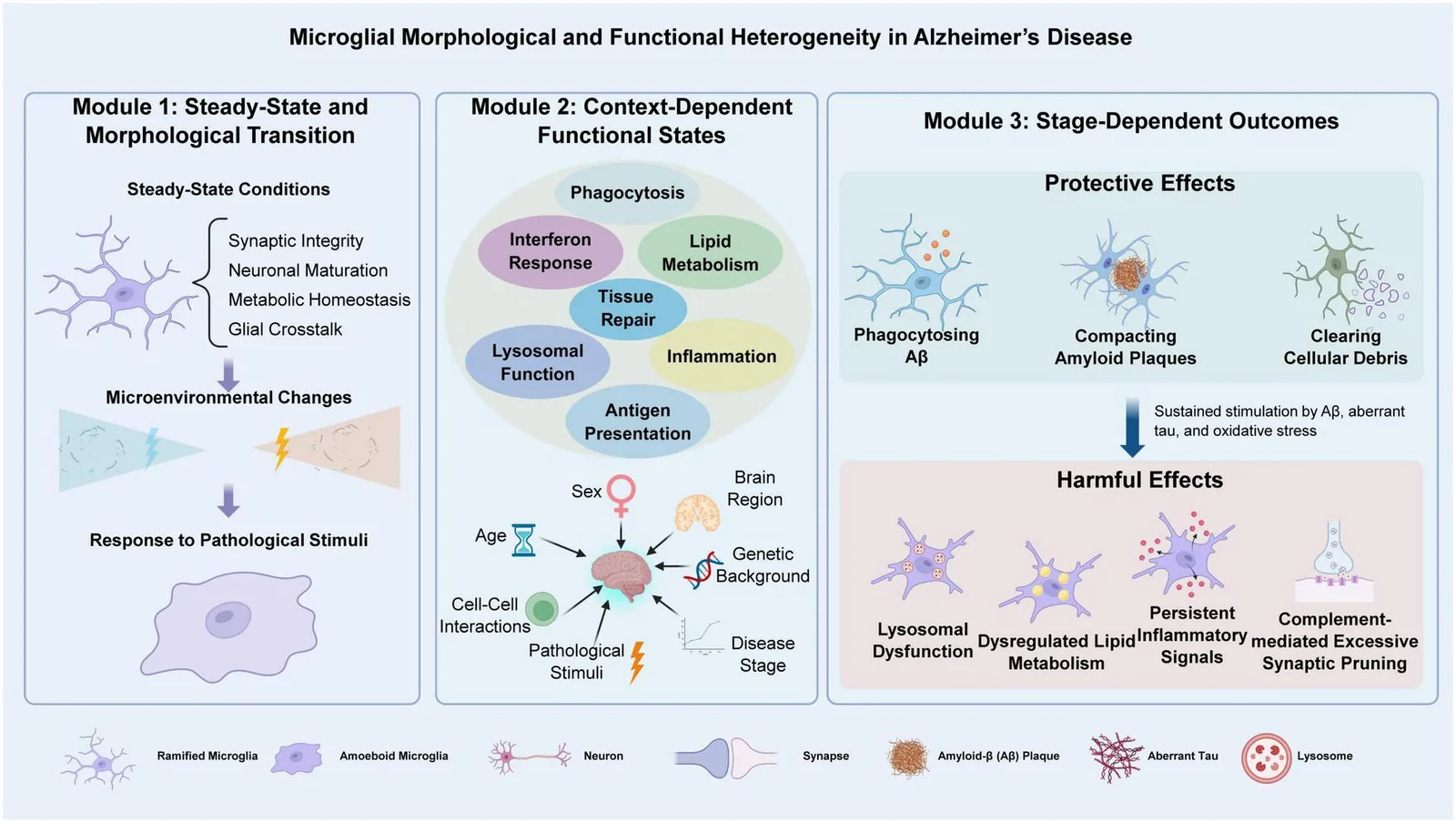
Microglial morphological and functional heterogeneity in Alzheimer’s disease. Under homeostatic conditions, ramified microglia continuously survey the brain microenvironment and contribute to synaptic integrity, neuronal maturation, metabolic homeostasis, and glial crosstalk. In response to pathological stimuli, microglia undergo morphological remodeling and adopt overlapping functional states involving phagocytosis, lysosomal activity, lipid metabolism, inflammation, interferon responses, antigen presentation, and tissue repair. These states are shaped by age, sex, brain region, genetic background, disease stage, pathological stimuli, and cell–cell interactions. In AD, microglia may initially promote Aβ clearance, plaque compaction, and cellular debris removal, whereas persistent stimulation by Aβ, pathological tau, and oxidative stress may shift the balance toward lysosomal dysfunction, dysregulated lipid metabolism, sustained inflammatory signaling, and complement-mediated excessive synaptic pruning. Aβ, amyloid-beta. Created in BioRender. Zhang, Y. (2026) https://BioRender.com/92mesj6.

As shown in Figure 1, under homeostatic conditions, ramified microglia continuously survey the brain microenvironment and contribute to synaptic integrity, neuronal maturation, metabolic homeostasis, and glial crosstalk. In response to pathological stimuli, microglia undergo morphological remodeling and adopt overlapping functional states involving phagocytosis, lysosomal activity, lipid metabolism, inflammation, interferon responses, antigen presentation, and tissue repair. These states are shaped by age, sex, brain region, genetic background, disease stage, pathological stimuli, and cell–cell interactions. In AD, microglia may initially promote Aβ clearance, plaque compaction, and cellular debris removal, whereas persistent stimulation by Aβ, pathological tau, and oxidative stress may shift the balance toward lysosomal dysfunction, dysregulated lipid metabolism, sustained inflammatory signaling, and complement-mediated excessive synaptic pruning.

## 3 The role of microglia in the neuroinflammatory pathogenesis of Alzheimer’s disease

### 3.1 Aβ-induced pro-inflammatory activation of microglia and amplification of the inflammatory cascade

Extracellular Aβ deposition is a defining neuropathological feature of AD and often emerges during the preclinical phase. However, Aβ accumulation should not be regarded as a surrogate for clinical disease progression. Substantial amyloid deposition may be present in cognitively unimpaired individuals, and amyloid positivity in the absence of tau pathology is associated with a substantially lower short-term risk of cognitive decline than combined amyloid and tau positivity. Moreover, tau-related biomarkers generally show closer associations with subsequent cognitive deterioration than amyloid burden (Bucci et al., 2021; Ossenkoppele et al., 2022; Sperling et al., 2024). Soluble Aβ peptides can self-associate into heterogeneous oligomeric and fibrillar assemblies that contribute to plaque formation. Nevertheless, soluble and deposited Aβ species can act as persistent pathological stimuli for microglia. Their multivalent conformations and exposed misfolded epitopes enable recognition by multiple pattern-recognition receptors on microglia, including Toll-like receptors (TLRs), the receptor for advanced glycation end products (RAGE) (Yan et al., 1996), scavenger receptors, and triggering receptor expressed on myeloid cells 2 (TREM2). Engagement of these receptors activates intracellular signaling programs that can shift microglia from homeostatic surveillance toward diverse reactive states.

CD14 is a GPI-anchored co-receptor that lacks an intracellular signaling domain. It is primarily responsible for capturing, enriching, and presenting Aβ at the cell surface (Kirschning et al., 1998; Reed-Geaghan et al., 2009). Through CD14-mediated mechanisms, Aβ further promotes the formation of TLR2/TLR4 recognition complexes. This is essential for cellular activation and the production of pro-inflammatory cytokines (Reed-Geaghan et al., 2009; Doens and Fernández, 2014). Concurrently, CD36, a class B scavenger receptor on microglial surfaces, can recognize and bind Aβ. CD36 then participates in Aβ adhesion, uptake, and endocytosis. Upon binding to CD36, Aβ promotes the assembly of a receptor complex between CD36 and TLR4/TLR6 on the cell membrane. This complex converts the Aβ recognition signal into an innate immune inflammatory signal. The complex further activates MyD88-dependent pathways, NF-κB, and MAPK signaling. It also promotes NADPH oxidase activation and ROS production. Ultimately, this process triggers the release of pro-inflammatory mediators such as TNF-α, IL-1β, and IL-6 (Doens and Fernández, 2014). RAGE can also bind to Aβ and exacerbate Aβ-related neuroinflammatory responses (Fang et al., 2010). It does this by amplifying oxidative stress, NF-κB activation, and inflammatory cascades.

Unlike the receptors mentioned above, which tend to initiate or amplify inflammation, TREM2 is more involved in regulating microglial migration, aggregation, phagocytosis, and the encapsulation of Aβ plaques. TREM2 deficiency impairs microglial ability to form a barrier around plaques and affects SYK-related signal transduction (Zhao et al., 2018; Wang S. et al., 2022). During early amyloid pathology, plaque-associated microglia may contribute to Aβ containment and clearance through phagocytosis, plaque compaction, and enzymatic degradation (Gerrits et al., 2021). However, persistent exposure to Aβ and other disease-associated signals, including pathological tau, oxidative stress, and cellular injury, can impair microglial clearance capacity and promote maladaptive inflammatory programs, thereby contributing to synaptic dysfunction and neuronal injury (Muzio et al., 2021; Pascoal et al., 2021; Wang et al., 2023). This leads to neurotoxicity and synaptic loss. Aβ-associated microglial activation is one of several context-dependent triggers of neuroinflammation. It serves as the foundation for subsequent inflammatory amplification (Pascoal et al., 2021; Wang et al., 2023).

### 3.2 Activation of the NLRP3 inflammasome

Activation of the NLRP3 inflammasome is a key molecular mechanism underlying the AD inflammatory response mediated by microglia (Milner et al., 2021). Inflammasomes are multiprotein signaling complexes composed of pattern recognition receptors (PRRs), adaptor proteins, and caspase-1. Within the brain, PRRs are predominantly found in microglia, macrophages, and astrocytes. Their expression is lower in oligodendrocytes. PRRs can be localized to the cell membrane or within the cytoplasm. There, they detect intracellular danger signals and induce a robust inflammatory response (Husemann et al., 2002; Kigerl et al., 2014; Li T. et al., 2022). Among inflammasomes involved in AD, NLRP3 has received the greatest research attention. When Aβ is phagocytosed by microglia, it can induce upstream danger signals. Such danger-associated signals include the efflux of K^+^ and Cl^–^, impaired mitochondrial function, ROS production, mtDNA leakage, and lysosomal disruption accompanied by the release of cathepsin B. These events promote NLRP3 activation. During activation, NLRP3 interacts specifically with NEK7. This binding is further enhanced upon inflammasome activation. It promotes NLRP3 oligomerization and the formation of an active complex. Subsequently, after oligomerization, NLRP3 interacts with apoptosis-associated speck-like protein containing a CARD (ASC) via PYD–PYD binding. ASC subsequently recruits pro-caspase-1, thereby promoting the assembly of the NLRP3 inflammasome complex (Huang et al., 2021; Vitale et al., 2023).

Upon activation of the NLRP3 inflammasome, pro-caspase-1 is cleaved and activated into caspase-1. Activated caspase-1 further cleaves the pro-cytokine precursors pro-IL-1β and pro-IL-18. This promotes their maturation into IL-1β and IL-18 (Martinon et al., 2002). In addition, caspase-1 mediates the cleavage of gasdermin D (GSDMD), generating an N-terminal domain that relocates to the cell membrane and forms membrane pores. This process induces pyroptotic cell death and promotes the extracellular release of IL-1β, IL-18, and other cellular contents. IL-1β is a potent pro-inflammatory mediator. IL-18 promotes IFN-γ production, which further amplifies the neuroinflammatory response. These cytokines also activate peripheral microglia and astrocytes. This perpetuates neuroinflammation and establishes a chronic inflammatory cycle. Activation of the NLRP3 inflammasome in microglia plays an essential role in driving the development and progression of AD. It amplifies the neuroinflammatory cascade, exacerbates synaptic dysfunction, and accelerates neuronal loss.

### 3.3 Mitochondrial dysfunction

Aβ deposition and other pathological factors can cause mitochondrial DNA damage, disrupting mitochondrial membrane permeability and altering metabolic activity and quality control. These mitochondrial changes promote microglial activation and enhance neuroinflammatory responses (Li Y. et al., 2022). Mitochondrial DNA polymorphisms in microglia regulate their activation status and phagocytic capacity for Aβ. When mitochondrial DNA is released from damaged cells, microglia recognize it via signaling pathways including TLR9, cytoplasmic cyclic GMP-adenylate synthase (cGAS)-stimulator of interferon genes (STING), and the NLRP3 inflammasome (Pinti et al., 2021). Oxidized mitochondrial DNA binds TLR9 on the endosomal membrane, activating the NLRP3 inflammasome and IFN pathways and significantly enhancing IFN-β expression (Liao et al., 2020). When stimulated by mitochondrial DNA, human microglia produce reactive oxygen species (ROS), activating the NF-κB pathway and inducing excessive production of pro-inflammatory cytokines. The local inflammatory environment is exacerbated, leading to cellular damage and tissue destruction (Simpson and Oliver, 2020).

At rest, microglia rely on mitochondrial oxidative phosphorylation for ATP. In inflammatory or stress conditions, energy supply becomes insufficient, causing mitochondrial dysfunction. To compensate, microglia increase glucose uptake via GLUT-1 and shift to anaerobic glycolysis to generate ATP rapidly and support immune function (Wang L. et al., 2019; Preeti et al., 2022). Studies show that inhibition of oxidative phosphorylation or electron transport chain activity induces microglial activation (Shaikh and Nicholson, 2009; Ye et al., 2016). Electron transport chain inhibitors cause morphological changes, ROS-mediated oxidative stress, activation of the MAPK/NF-κB pathway, inflammasome activation, overproduction of pro-inflammatory cytokines, and accumulation of damaged mitochondria. This cascade leads to microglial dysfunction and apoptosis (Shaikh and Nicholson, 2009; Ye et al., 2016). Microglial mitochondrial dysfunction can trigger neuroinflammatory responses, leading to neuronal degeneration, thereby contributing to AD pathogenesis.

### 3.4 Dysregulation of phagocytosis and impaired Aβ clearance

Impairment of microglial phagocytosis and defective Aβ clearance play key roles in maintaining chronic neuroinflammatory responses in AD. In humans and in AD transgenic models, activated microglia are highly correlated with senile plaques (Zuroff et al., 2017). When microglia respond to neuronal injury or other pathological stimuli, their cell bodies and processes migrate to the injury site, where they clear fibrillar and soluble Aβ through phagocytosis and proteolytic processes (Heneka et al., 2015). In addition to Aβ clearance, they also initiate local immune responses to control pathological damage (Paresce et al., 1996; Mosher and Wyss-Coray, 2014). This process is mediated by multiple receptors on microglial surfaces—including TLRs, scavenger receptor 1, CD36, TREM2, and CD33—which play crucial roles in Aβ recognition and phagocytosis (Zuroff et al., 2017).

However, persistent Aβ deposition and a prolonged inflammatory microenvironment disrupt these protective functions over time, leading to gradual functional failure or dysregulation of microglia. Consequently, their phagocytic ability is reduced, leading to the development of chronic neuroinflammation. As a result, activated microglia continuously generate ROS, immunomodulatory factors, pro-inflammatory cytokines such as IL-1β, IL-6, TNF-α, and TGF-β, and chemokines including macrophage inflammatory protein, monocyte chemoattractant protein-1, CCL3, and CCL5 (Xia et al., 1998; Wyss-Coray and Mucke, 2000; Wyss-Coray and Rogers, 2012). The upregulation of these inflammatory mediators has been associated with marked neurotoxicity (Wyss-Coray and Mucke, 2002; Takeuchi et al., 2006) as well as exacerbated Aβ pathology in particular brain regions of human AD cases and APP/PS1 transgenic mice (López-González et al., 2015). Overall, microglia in AD should not simply be categorized as neuroprotective or neurotoxic. The main abnormality is not just “overactivation” but rather a disrupted balance between protective and damaging functions. This disruption leads to reduced phagocytic capacity and persistent inflammation, ultimately driving the disease toward a chronic, progressive course.

### 3.5 Complement-mediated abnormal synaptic pruning

The complement system is a crucial component of the innate immune response. Complement molecules like complement component 1q (C1q) act as pattern recognition molecules, helping recognize and clear pathogens, damaged tissues, aggregated proteins, and toxic metabolic waste (Köhl, 2006, p. 157; Holers, 2014). Beyond peripheral immunity, complement proteins play roles in brain development, aging, and neurological diseases (Lee et al., 2019). Microglia exhibit expression of nearly the full range of classical complement components and receptors, such as C1qR, CR3, C3aR, and C5aR (Veerhuis et al., 2011; Laumonnier et al., 2017). Complement and microglia together drive synaptic pruning during neurodevelopment, aging, and neurodegenerative disease. Microglia clear synapses tagged by complement (Stephan et al., 2012). Because microglia and complement are central to synaptic pruning, complement-mediated microglial phagocytosis may promote Alzheimer’s pathology (Tremblay et al., 2011; Crehan et al., 2012). C1q and C3 fragments can mark synapses for removal. Microglia, equipped with complement receptors, then clear these synapses, leading to excess pruning. Microglia and complement disrupt synaptic transmission and cause early synaptic loss in the presence of Aβ oligomers. Microglia phagocytose synapses before visible Aβ plaques, responding to complement factors C1q, C3, and CR3. Inhibition of C3, C1q, or CR3 can protect against early synaptic loss in *APP/PS1* mice (Hong et al., 2016). The complement system and NLRP3 inflammasome also interact, together regulating TLR-driven immune and inflammatory responses. TLR4 can start inflammation in peripheral tissues and activate the NLRP3 inflammasome in the CNS (Yang et al., 2020). As Aβ accumulates, the “Aβ–TLR4–NLRP3 inflammasome–IL-1β” pathway in microglia is repeatedly triggered, fueling neuroinflammation and neurodegeneration in Alzheimer’s disease (Yang et al., 2020).

### 3.6 Bidirectional interactions between microglia-mediated neuroinflammation and tau pathology

Tau pathology should not be regarded solely as a downstream consequence of Aβ deposition; rather, it represents a key pathological process closely associated with neurodegeneration and clinical progression in AD. Under pathological conditions, tau undergoes abnormal phosphorylation, conformational changes, and misfolding, leading to the formation of soluble oligomers and fibrillar aggregates that ultimately constitute neurofibrillary tangles. As tau pathology extends from the medial temporal lobe to neocortical association regions, its regional distribution and pathological burden are generally more closely associated with synaptic dysfunction, neuronal loss, brain atrophy, and cognitive impairment than the overall burden of fibrillar Aβ. Longitudinal imaging studies have further demonstrated that baseline regional tau-PET signals predict subsequent brain atrophy and decline in specific cognitive domains, whereas baseline amyloid burden shows substantially weaker associations with these changes, with some studies reporting no significant relationship (Bucci et al., 2021; Lagarde et al., 2022).

Microglia-mediated neuroinflammation and tau pathology interact in a bidirectional and context-dependent manner. Pro-inflammatory mediators released by reactive microglia and astrocytes, including IL-1β and TNF-α, can promote abnormal tau phosphorylation and misfolding while exacerbating neuronal stress (Ising et al., 2019; Ou et al., 2021; Chen and Yu, 2023). Consistent with these findings, inhibition of IL-1 signaling attenuated tau pathology and improved cognitive function in AD mouse models (Kitazawa et al., 2011). Human PET studies have further indicated that tau burden and microglial activation provide complementary prognostic information regarding cognitive decline and exhibit spatial and temporal coupling across different Braak stages (Malpetti et al., 2020; Pascoal et al., 2021). These findings suggest that neuroinflammation may contribute to the accumulation and propagation of pathological tau; however, this process cannot be reduced to a simple linear cascade initiated solely by Aβ.

On the other hand, neurons that are damaged or under stress can release tau into the extracellular space, where it is subsequently recognized and internalized by microglia. Microglial uptake of tau may initially contribute to its clearance, whereas persistent exposure to aggregated tau can activate maladaptive innate immune signaling pathways. Microglial recognition of tau through the polyglutamine-binding protein 1–cyclic GMP-AMP synthase–stimulator of interferon genes (PQBP1–cGAS–STING) pathway can induce inflammatory responses (Jin et al., 2021). In addition, microglial NF-κB signaling can promote the processing and release of seeding-competent tau species, thereby facilitating the intercellular propagation of tau pathology. Genetic or pharmacological inhibition of NF-κB reduces tau propagation and neurotoxicity in tauopathy models (Wang C. et al., 2022). Internalized tau aggregates can also activate the NLRP3–ASC inflammasome, whereas inhibition of NLRP3 or genetic deletion of ASC attenuates both seeded and spontaneous tau pathology *in vivo* (Stancu et al., 2019).

Collectively, pathological tau can induce maladaptive microglial responses, while sustained neuroinflammation can further promote abnormal tau accumulation, intercellular propagation, and neuronal injury, thereby establishing a potential self-reinforcing cycle (Chen and Yu, 2023). Nevertheless, microglia also possess the capacity to internalize and clear tau, and their effects may vary according to disease stage, cellular state, and the local brain microenvironment. Therapeutic strategies should therefore selectively target disease-promoting inflammatory pathways rather than broadly or indiscriminately suppressing microglial function.

**FIGURE 2:**
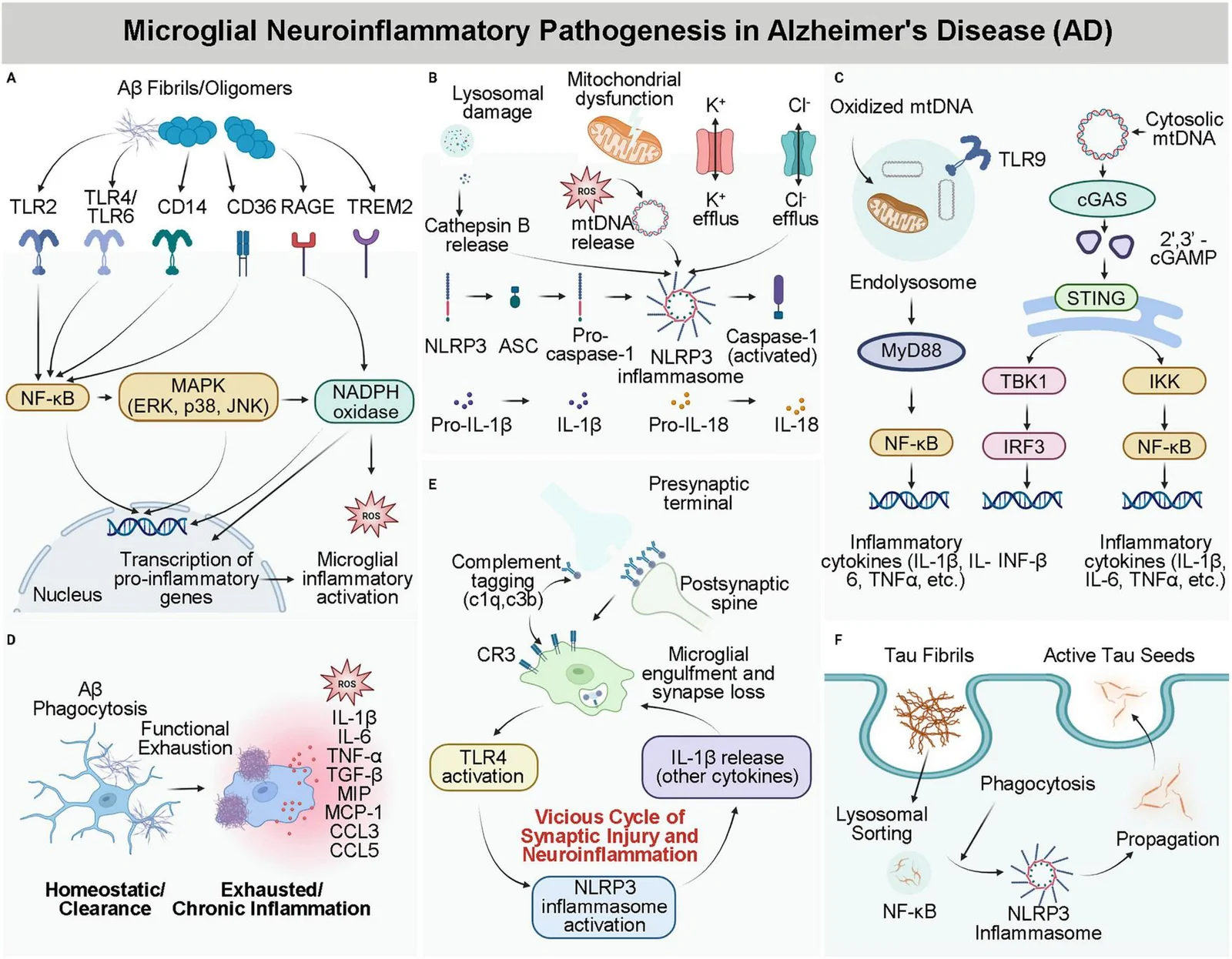
Microglial neuroinflammatory pathogenesis in Alzheimer’s disease (AD). **(A)** Aβ is recognized by microglial receptors like TLR2, TLR4/TLR6, CD14, CD36, RAGE, and TREM2. This recognition triggers pathways including NF-κB, MAPK, and NADPH oxidase that induce pro-inflammatory gene expression, ROS production, and inflammation. **(B)** Lysosomal damage, cathepsin B release, mitochondrial dysfunction, mtDNA release, and K^+^/Cl^–^ efflux contribute to NLRP3 inflammasome assembly, activating caspase-1 and leading to the maturation and release of IL-1β and IL-18. **(C)** Oxidized mtDNA activates NF-κB via TLR9–MyD88. Cytoplasmic mtDNA induces inflammation through cGAS–STING–TBK1/IRF3 and IKK–NF-κB pathways. **(D)** Ongoing Aβ and tau stimulation exhausts microglial function, leading to chronic inflammation with increased levels of IL-1β, IL-6, TNF-α, CCL3, and CCL5. **(E)** Complement C1q/C3 marks damaged synapses, driving CR3-mediated excessive synaptic pruning. The complement system and NLRP3 inflammasome jointly regulate TLR-mediated immune responses. TLR4 can activate NLRP3 in the CNS. As Aβ aggregates, the “Aβ–TLR4–NLRP3 inflammasome–IL-1β” axis is continually activated, amplifying neuroinflammation. **(F)** Tau fibrils and seeds are taken up by microglia and processed in lysosomes; impaired clearance allows tau seeds to further promote inflammasome activation and the spread of tau pathology. AD, Alzheimer’s disease; Aβ, amyloid-β; TLR2, Toll-like receptor 2; TLR4, Toll-like receptor 4; TLR6, Toll-like receptor 6; CD14, cluster of differentiation 14; CD36, cluster of differentiation 36; RAGE, receptor for advanced glycation end products; TREM2, triggering receptor expressed on myeloid cells 2; NF-κB, nuclear factor kappa B; MAPK, mitogen-activated protein kinase; ERK, extracellular signal-regulated kinase; JNK, c-Jun N-terminal kinase; NADPH oxidase, nicotinamide adenine dinucleotide phosphate oxidase; ROS, reactive oxygen species; mtDNA, mitochondrial DNA; NLRP3, NOD-like receptor family pyrin domain-containing 3; ASC, apoptosis-associated speck-like protein containing a CARD; IL-1β, interleukin-1β; IL-6, interleukin-6; IL-18, interleukin-18; TNF-α, tumor necrosis factor-α; TGF-β, transforming growth factor-β; MCP-1, monocyte chemoattractant protein-1; MIP, macrophage inflammatory protein; CCL3, C-C motif chemokine ligand 3; CCL5, C-C motif chemokine ligand 5; TLR9, Toll-like receptor 9; cGAS, cyclic GMP-AMP synthase; 2′,3′-cGAMP, 2′,3′-cyclic GMP-AMP; STING, stimulator of interferon genes; MyD88, myeloid differentiation primary response 88; TBK1, TANK-binding kinase 1; IKK, IκB kinase; IRF3, interferon regulatory factor 3; IFN-β, interferon-β; C1q, complement component 1q; C3b, complement component 3b; CR3, complement receptor 3. Created in BioRender. Zhang, Y. (2026) https://BioRender.com/0ezozbb.

Figure 2 summarizes the major microglia-mediated inflammatory mechanisms in AD, showing how Aβ accumulation, mitochondrial damage, complement activation, and tau pathology converge to promote neuronal injury. These pathological processes provide the mechanistic basis for the microglia-related therapeutic targets discussed below. The mechanisms, representative interventions, and translational evidence for these targets are summarized in Table 1.

**TABLE 1:** Microglia-related therapeutic targets in Alzheimer’s disease: mechanisms, representative interventions, and translational evidence.

Therapeutic target / pathway	Representative intervention(s)	Proposed microglial mechanism	Key evidence in AD models or clinical studies	Development status and main limitation
TREM2 agonism	AL002 / AL002c	Promotes TREM2 clustering and TYROBP/DAP12-SYK-PI3K-AKT signaling, supporting plaque-associated microglial recruitment, survival, metabolic fitness, and phagocytic or plaque-containment responses.	AL002c increased microglial proliferation and reduced fibrillar plaques and neuritic dystrophy in human-TREM2 5xFAD mice (Wang et al., 2020). In the phase 2 INVOKE-2 trial (*n* = 381), AL002 produced sustained CNS target engagement but did not improve CDR-SB versus placebo (Mummery et al., 2026).	Phase 2 completed; negative efficacy trial. ARIA-like MRI changes were frequent. Target engagement did not translate into clinical benefit.
TREM2 agonism with BBB transport	Ab18 TVD-Ig/αTfR	Combines tetravalent TREM2 clustering with TfR-mediated transcytosis to increase brain exposure and enhance microglial metabolic and plaque-associated responses.	The construct achieved more than tenfold higher brain exposure than its non-TfR counterpart and increased plaque-associated microglia and Aβ phagocytosis while reducing amyloid burden, tau phosphorylation, synaptic injury, and cognitive deficits in AD mouse models (Zhao et al., 2022).	Preclinical only. Human safety, central pharmacodynamics, and efficacy remain unknown; effects may depend on disease stage and baseline TREM2 expression.
CD33 (SIGLEC3) inhibitory signaling	HuM195 and HuM195 scFv	Induces CD33 internalization and degradation, relieving ITIM-SHP-1-mediated inhibition of Aβ uptake; may also increase IL-33 secretion.	HuM195 and its scFv enhanced Aβ42 phagocytosis by human microglia and monocytes (Wong et al., 2024). Genetic or gene-therapy-mediated reduction of *CD33* decreased amyloid accumulation and neuroinflammation in AD models (Griciuc et al., 2020), and the benefit of *CD33* deletion depended on intact TREM2 signaling (Griciuc et al., 2019).	Cellular and animal evidence only. Translation is complicated by CD33 isoform-specific effects, ligand context, and functional interaction with TREM2.
CD33 (SIGLEC3) targeting	AL003	Anti-CD33 monoclonal antibody intended to relieve inhibitory myeloid signaling and enhance microglial activity.	AL003 entered phase 1 clinical evaluation, but no evidence of cognitive or functional efficacy in AD is available (Hampel et al., 2020).	Clinical development did not establish efficacy; no cognitive or functional benefit has been demonstrated.
P2X7 receptor	Brilliant Blue G; UB-ALT-P2	Blocks ATP-gated P2X7 signaling upstream of K+ efflux, Ca2+ influx, oxidative stress, and NLRP3 inflammasome activation.	Brilliant Blue G reduced GSK-3β activity and hippocampal amyloid burden in J20 mice (Diaz-Hernandez et al., 2012). The orally bioavailable, brain-penetrant UB-ALT-P2 improved memory and reduced Aβ, phosphorylated tau, oxidative stress, and inflammatory markers in 5xFAD mice (Turcu et al., 2026).	Preclinical. Early antagonists have selectivity and exposure limitations; the UB-ALT-P2 report remains a non-peer-reviewed preprint.
NLRP3 inflammasome	MCC950; dapansutrile (OLT1177); VEN-02XX	Inhibits NLRP3 assembly and downstream caspase-1 activation, IL-1β/IL-18 maturation, ASC-speck amplification, and pyroptotic signaling.	MCC950 and OLT1177 improved cognition or synaptic plasticity and reduced amyloid-related pathology in APP/PS1 mice (Dempsey et al., 2017; Lonnemann et al., 2020). Post-symptomatic VEN-02XX improved learning and reduced reactive gliosis, phosphorylated tau, and plasma neurofilament light in 5xFAD/Rubicon-knockout mice (Auger et al., 2025). Conversely, *Nlrp3* or microglial *Gsdmd* deficiency did not improve aggressive P301S tauopathy (Paesmans et al., 2024).	Preclinical and model-dependent. Chronic blockade may impair host defense and tissue repair; AD-specific clinical efficacy has not been established.
cGAS signaling	TDI-6570; TDI-8246; microglial *Cgas* deletion	Blocks cytosolic DNA sensing and cGAMP production, thereby reducing STING-TBK1-IRF3 and NF-κB-driven type I interferon responses.	TDI-6570 suppressed interferon signaling and restored MEF2C-associated synaptic and cognitive measures in P301S mice; TDI-8246 reduced CXCL10 and CCL5 in tau-stimulated human iPSC-derived microglia (Udeochu et al., 2023). Inducible microglia-specific *Cgas* deletion limited plaques, inflammasome activation, and cognitive impairment in 5xFAD mice (He et al., 2025).	Preclinical. Brain penetration, microglial selectivity, and the risk of compromising antiviral DNA sensing require evaluation.
STING signaling	H-151	Inhibits STING activation, type I interferon signaling, and inflammatory convergence with the NLRP3 inflammasome.	H-151 reduced Aβ deposition, gliosis, and cognitive deficits in 5xFAD mice (Xie et al., 2023). In *AppNL-G-F*/hTau mice, it also reduced Aβ burden, tau phosphorylation, microglial synapse engulfment, and memory impairment (Chung et al., 2024).	Preclinical. Optimal treatment duration, CNS exposure, and immunological safety are unresolved.
CSF1R: partial inhibition	GW2580; edicotinib (JNJ-40346527); PLX3397	Restricts CSF1R-dependent microglial proliferation and may partially reset population composition or phagocytic function without complete depletion.	GW2580 improved synaptic integrity and memory without reducing plaque number in APP/PS1 mice (Olmos-Alonso et al., 2016). Edicotinib attenuated tau-associated neurodegeneration in P301S mice (Mancuso et al., 2019). Short-term PLX3397 increased phagocytic clearance of terminal Aβ and restored LTP in acute hippocampal slices (Piccioni et al., 2024).	Preclinical. Evidence supports stage-, dose-, and model-dependent effects, not a general anti-inflammatory action.
CSF1R: depletion and repopulation	PLX5622; PLX3397	Produces profound microglial depletion followed by niche repopulation after withdrawal, altering the size and composition of the microglial population.	Pre-plaque depletion reduced parenchymal plaque initiation but redirected Aβ toward cerebral vessels (Spangenberg et al., 2019). Depletion after plaque formation reduced plaque compaction and worsened neuritic injury (Baligács et al., 2024). Partial depletion and repopulation produced only subtle or transient effects, and late plaque-associated microglia were relatively CSF1R-resistant (Le et al., 2024).	Preclinical. Potential loss of homeostatic and plaque-barrier functions and possible cerebral amyloid angiopathy risk favor partial or pulsed dosing.
Complement C1q	Anti-C1q antibodies; ANX005; microglial C1q deletion	Limits classical complement initiation, complement tagging of vulnerable synapses, and subsequent microglial synapse engulfment.	C1q antibodies rescued tau-induced synapse loss in P301S mice (Dejanovic et al., 2018). Young-adult microglial C1q deletion reduced synaptic engulfment and partially improved cognition but also reduced amyloid compaction (Petrisko et al., 2026). ANX005 inhibited C1q in serum and CSF in Guillain-Barré syndrome, demonstrating human pharmacological feasibility rather than AD efficacy (Mohammad et al., 2025).	AD evidence remains preclinical. Long-term blockade may impair debris clearance, plaque compaction, and host defense.
Complement C5aR1	PMX205	Reduces C5a-driven microglial and astrocytic reactivity while potentially preserving upstream complement opsonization and clearance.	PMX205 reduced VGLUT1-positive synapse loss and improved LTP in an age- and brain-region-dependent manner (Gomez-Arboledas et al., 2024). It also suppressed detrimental glial transcriptional programs and preserved short-term memory in female Arctic48 mice without changing hippocampal plaque burden (Schartz et al., 2024).	Preclinical only. Sex, age, region, and disease-stage dependence require prospective validation.
Nonselective TNF blockade	Etanercept; adalimumab	Systemically blocks soluble and transmembrane TNF without microglial specificity; CNS penetration is limited.	A 24-week phase 2 trial of subcutaneous etanercept in 41 patients with mild-to-moderate AD primarily established short-term tolerability and did not show statistically significant benefits in cognition, behavior, or global function (Butchart et al., 2015). No published AD randomized controlled efficacy trial of adalimumab is available; the cited evidence comprises a case report, a retrospective association study, and an animal study (Camargo et al., 2015; Park et al., 2019; Zhou et al., 2020).	Clinical evidence negative or insufficient. Systemic immunosuppression, poor CNS exposure, and simultaneous blockade of tmTNF limit interpretability.
Selective soluble TNF inhibition	XPro1595	Forms inactive heterotrimers with endogenous soluble TNF, preferentially sparing tmTNF-TNFR2 signaling associated with immune regulation and repair.	Peripheral XPro1595 altered brain immune-cell profiles, reduced amyloid plaque load, and rescued impaired LTP in 5xFAD mice (MacPherson et al., 2017).	Preclinical AD evidence. Molecular-form selectivity does not confer microglial specificity, and clinical efficacy in AD is unproven.
RIPK1 kinase	SAR443060 (DNL747); SAR443820 (DNL788)	Inhibits RIPK1 kinase-dependent inflammatory and cell-death signaling while aiming to preserve kinase-independent scaffolding functions.	RIPK1 inhibition reduced maladaptive microglial programs, lysosomal dysfunction, and amyloid burden in preclinical models (Ofengeim et al., 2017). Phase I/Ib SAR443060 achieved CSF exposure and peripheral pRIPK1 inhibition but provided no evidence of clinical efficacy and was discontinued after long-term nonclinical toxicology findings (Vissers et al., 2022). SAR443820 showed CSF exposure and peripheral target engagement in healthy participants (Hincelin-Mery et al., 2024).	Early clinical development. Adequate target inhibition within human microglia is unproven; SAR443060 toxicity appears compound-specific rather than an established class effect.

Ab, antibody; AD, Alzheimer’s disease; AKT, protein kinase B; APP/PS1, amyloid precursor protein/presenilin 1; Aβ, amyloid-β; Aβ42, amyloid-β 1–42; ARIA, amyloid-related imaging abnormalities; ASC, apoptosis-associated speck-like protein containing a caspase recruitment domain; ATP, adenosine triphosphate; BBB, blood–brain barrier; C1q, complement component 1q; C5aR1, complement C5a receptor 1; CCL5, C–C motif chemokine ligand 5; CD33, cluster of differentiation 33; CDR-SB, Clinical Dementia Rating–Sum of Boxes; cGAMP, cyclic GMP–AMP; cGAS, cyclic GMP–AMP synthase; CNS, central nervous system; CSF, cerebrospinal fluid; CSF1R, colony-stimulating factor 1 receptor; CXCL10, C–X–C motif chemokine ligand 10; DAP12, DNAX-activating protein of 12 kDa; GSDMD, gasdermin D; GSK-3β, glycogen synthase kinase 3β; IL, interleukin; iPSC, induced pluripotent stem cell; IRF3, interferon regulatory factor 3; ITIM, immunoreceptor tyrosine-based inhibitory motif; LTP, long-term potentiation; MEF2C, myocyte enhancer factor 2C; MRI, magnetic resonance imaging; NF-κB, nuclear factor-κB; NLRP3, NOD-like receptor family pyrin domain-containing 3; P2X7, purinergic P2X receptor 7; PI3K, phosphoinositide 3-kinase; pRIPK1, phosphorylated RIPK1; RIPK1, receptor-interacting serine/threonine-protein kinase 1; scFv, single-chain variable fragment; SHP-1, Src homology 2 domain-containing protein tyrosine phosphatase 1; SIGLEC3, sialic acid-binding immunoglobulin-like lectin 3; STING, stimulator of interferon genes; SYK, spleen tyrosine kinase; TBK1, TANK-binding kinase 1; TfR, transferrin receptor; TREM2, triggering receptor expressed on myeloid cells 2; TNF, tumor necrosis factor; tmTNF, transmembrane TNF; TNFR, tumor necrosis factor receptor; TVD-Ig, tetra-variable domain immunoglobulin; TYROBP, transmembrane immune signaling adaptor; VGLUT1, vesicular glutamate transporter 1; 5xFAD, five familial Alzheimer’s disease mutation mouse model; αTfR, anti-transferrin receptor.

## 4 Therapeutic targets associated with microglia in AD

### 4.1 Therapeutic modulation of TREM2 and CD33 receptor signaling

TREM2 is an immune receptor predominantly expressed by microglia and is closely involved in CNS innate immunity, lipid sensing, and the regulation of neuroinflammation. The rare TREM2 R47H variant impairs ligand recognition, microglial metabolic adaptation, and responses to pathological injury, thereby substantially increasing the risk of AD and highlighting the importance of TREM2 in maintaining CNS immune homeostasis (Guerreiro et al., 2013; Lu et al., 2026). Given its central role in neuroimmune interactions, TREM2 has emerged as a compelling target for modulating innate immunity in AD. TREM2 recognizes phospholipids, lipoproteins, and damage-associated lipids, and apolipoprotein E (APOE) is also one of its ligands. Following ligand binding, TREM2 signals through the adaptor protein TYROBP/DAP12 and downstream pathways involving SYK and PI3K–AKT, thereby regulating microglial survival, phagocytosis, and energy metabolism (Atagi et al., 2015; Wang et al., 2015; Ulland et al., 2017). In models of Aβ deposition, TREM2 deficiency impairs microglial recruitment to plaques and plaque compaction, while increasing plaque-associated neuritic damage. By contrast, intact TREM2 signaling supports microglial metabolic fitness and the acquisition of plaque-associated response states (Wang et al., 2015; Ulland et al., 2017). TREM2–APOE signaling also contributes to the transition of microglia from a homeostatic state to a disease-associated transcriptional state, characterized by the downregulation of homeostatic genes and the upregulation of genes involved in lipid metabolism, phagocytosis, and lysosomal function. Although this transition may enhance microglial recruitment to Aβ plaques, plaque containment, and the clearance of cellular debris, sustained or inappropriately timed activation may result in the loss of homeostatic functions, prolonged inflammatory responses, and neuronal injury (Keren-Shaul et al., 2017; Krasemann et al., 2017). Thus, the disease-associated microglial state should not be regarded as uniformly neuroprotective.

Based on these preclinical findings, several TREM2-agonistic antibodies have entered preclinical or clinical development. AL002 is a TREM2 agonist antibody. In the phase 1 INVOKE-1 study, a single intravenous dose of AL002 decreased soluble TREM2 levels in the cerebrospinal fluid and increased downstream biomarkers such as osteopontin, indicating CNS exposure and target engagement (Wang et al., 2020). However, these pharmacodynamic effects did not translate into clinical benefit. The completed and published phase 2 randomized INVOKE-2 trial was negative: although AL002 achieved CNS target engagement, it failed to meet its primary efficacy endpoint (Mummery et al., 2026). This negative result does not invalidate the biological relevance of TREM2 but indicates that target engagement alone is insufficient to modify the clinical course of AD. Future studies may require biomarker-guided patient stratification, optimization of dose and therapeutic timing, and careful evaluation of TREM2 modulators in combination with anti-Aβ or anti-tau therapies, provided that such combinations are supported by adequate mechanistic evidence. In addition to AL002, Zhao et al. developed Ab18 TVD-Ig/αTfR, a tetravalent, TREM2-agonistic, transferrin receptor (TfR)-targeting bispecific antibody. Its tetravalent TREM2-binding configuration enhances receptor clustering and downstream signaling, whereas engagement of TfR facilitates receptor-mediated transcytosis across the blood–brain barrier and improves delivery to the brain parenchyma. In 5xFAD mice, Ab18 TVD-Ig/αTfR achieved more than a tenfold increase in brain exposure compared with its non-TfR-targeting counterpart. Treatment increased microglial recruitment to Aβ plaques and Aβ phagocytosis, reduced amyloid burden, preserved synaptic and neuronal markers, decreased tau hyperphosphorylation, and ameliorated cognitive deficits (Zhao et al., 2022). Nevertheless, these findings are restricted to AD mouse models, and no human safety, pharmacodynamic, or clinical efficacy data are currently available. TREM2-dependent modulation of microglial activity therefore remains a biologically plausible therapeutic strategy; however, whether it can delay the onset or progression of AD in humans remains to be established.

*CD33* (Siglec-3) is another important AD susceptibility gene that encodes a member of the sialic acid-binding immunoglobulin-like lectin family. The encoded protein is a type I transmembrane receptor containing cytoplasmic immunoreceptor tyrosine-based inhibitory motifs (ITIMs) and regulates immune responses in myeloid cells (Eskandari-Sedighi et al., 2023). Within the brain parenchyma, CD33 is predominantly expressed by microglia. Several postmortem human studies have reported increased CD33 expression and greater numbers of CD33-positive microglia in AD brains, which positively correlated with insoluble Aβ42 levels and amyloid plaque burden. However, increases in total CD33 protein have not been consistently replicated across postmortem cohorts, suggesting that CD33 expression may be influenced by genetic background, disease stage, and microglial state. CD33 upregulation should therefore not be regarded as a universal feature of all individuals with AD (Griciuc et al., 2013; Walker et al., 2015). Notably, the pathological effects of CD33 are strongly isoform-dependent. The full-length CD33M isoform, which is associated with increased AD risk, recruits phosphatases such as SHP-1 via its cytoplasmic ITIMs, thereby suppressing microglial phagocytic signaling. Recent work further demonstrated that clusterin (CLU) and CLU–Aβ complexes can bind CD33M, induce ITIM phosphorylation and SHP-1 recruitment, and consequently suppress Aβ phagocytosis and amyloid plaque clearance (Dodd et al., 2026). In 5xFAD mice expressing human CD33 isoforms, CD33M increased cerebral Aβ levels and the abundance of diffuse plaques, attenuated plaque-associated microglial responses, and exacerbated plaque-associated neuritic injury. By contrast, the protective short isoform CD33m enhanced microglia–plaque interactions and plaque compaction while reducing neuritic pathology (Eskandari-Sedighi et al., 2024). These findings indicate that CD33 should not be viewed simply as a deleterious receptor; rather, its effects depend on the specific isoform, ligand environment, and disease stage. Consistent with this concept, CD33 inhibits microglial Aβ uptake in cellular models, whereas reducing or genetically deleting *CD33* decreases amyloid plaque burden in APP/PS1 mice (Griciuc et al., 2020).

Based on these mechanisms, the relief of CD33-mediated inhibitory signaling has emerged as a potential therapeutic strategy. The anti-CD33 antibody HuM195 and its single-chain variable fragment (scFv) induce CD33 internalization and degradation, thereby enhancing Aβ42 phagocytosis by human microglia and monocytes. HuM195 can also transiently induce CD33 dimerization and phosphorylation and promote IL-33 secretion, providing an experimental rationale for improving Aβ clearance through the functional reprogramming of microglia (Wong et al., 2024). Nevertheless, these findings are derived primarily from cellular and animal studies and do not demonstrate improved cognitive outcomes in patients with AD. Although the CD33-blocking antibody AL003 showed evidence of target engagement in a phase 1 study, its development program was subsequently discontinued because of insufficient evidence of an effect on pharmacodynamic biomarkers. No data currently support its clinical efficacy (Hampel et al., 2020). Furthermore, CD33 and TREM2 do not operate as independent regulatory pathways. In 5xFAD mice, the reductions in Aβ burden, improvements in cognition, and transcriptional remodeling of microglia induced by *CD33* deletion depended on intact TREM2 signaling. When *TREM2* was simultaneously deleted, the protective effects of *CD33* deficiency were markedly attenuated, suggesting that TREM2 may function downstream of CD33 within this regulatory network (Griciuc et al., 2019). Accordingly, the therapeutic response to CD33 targeting may be jointly influenced by TREM2 function, TREM2 risk variants, the relative abundance of CD33 isoforms, and the pre-existing functional state of microglia.

### 4.2 Targeting danger-signal sensing and pathological amplification: P2X7-mediated NLRP3 inflammasome signaling and the cGAS–STING pathway

In the brains of patients with AD, Aβ stimulation can induce microglial ATP release and establish an autocrine oxidative stress signal (Kim et al., 2007); meanwhile, mitochondrial stress induced by Aβ or pathological tau can cause the aberrant leakage of mitochondrial DNA (mtDNA) and other self-DNA into the microglial cytosol, thereby activating cGAS–STING signaling (Udeochu et al., 2023; Chung et al., 2024). Microglia respond to high concentrations of extracellular ATP through the P2X7–NLRP3 axis and sense ectopic cytosolic DNA through the cGAS–STING axis; the latter drives type I interferon responses and can engage in crosstalk with the NLRP3 inflammasome (Udeochu et al., 2023; Xie et al., 2023). In AD models, genetic ablation or pharmacological inhibition of NLRP3, cGAS, or STING can attenuate Aβ- or tau-associated inflammation, synaptic damage, and cognitive impairment. Therefore, rather than merely functioning downstream of Aβ or tau pathology, these two pathways serve as critical “pathological amplifiers” linking cellular damage, innate immune activation, and neurodegenerative changes (Ising et al., 2019; Xie et al., 2023).

P2X7 is a ligand-gated, nonselective cation channel with relatively low affinity for ATP and therefore generally requires high concentrations of extracellular ATP for full activation. In tissues derived from patients with Alzheimer’s disease (AD) and in transgenic mouse models, P2X7 expression is upregulated and enriched in reactive microglia surrounding Aβ plaques (Parvathenani et al., 2003; McLarnon et al., 2006; Sanz et al., 2009). Opening of the P2X7 channel induces K^+^ efflux and Ca^2 +^ influx and can promote NADPH oxidase-dependent production of superoxide anions and other reactive oxygen species (ROS) (Parvathenani et al., 2003; McLarnon et al., 2006). Among these events, a reduction in intracellular K^+^ is a well-established proximal signal for NLRP3 activation (Muñoz-Planillo et al., 2013). Subsequently, NLRP3, ASC, and pro-caspase-1 assemble into the inflammasome complex, leading to caspase-1 activation, maturation of IL-1β and IL-18, and gasdermin D (GSDMD) cleavage, which promotes membrane-pore formation and pyroptosis (Shi et al., 2015; Swanson et al., 2019). Aβ-induced inflammatory responses in microglia depend on P2X7 expression, whereas genetic deletion of *Nlrp3* or *Casp1* attenuates neuroinflammation, amyloid pathology, and cognitive impairment in APP/PS1 mice (Sanz et al., 2009; Heneka et al., 2013). More importantly, ASC specks released by microglia can directly promote the extracellular cross-seeding of Aβ, while NLRP3 activation can drive tau hyperphosphorylation by modulating tau kinases and phosphatases, thereby establishing a positive feedback loop involving “Aβ or tau–inflammasome–protein aggregation” (Venegas et al., 2017; Ising et al., 2019). Nevertheless, P2X7 represents only one of the major upstream pathways leading to NLRP3 activation; Aβ-mediated Toll-like receptor (TLR) signaling, lysosomal rupture, and mitochondrial ROS can also activate the NLRP3 inflammasome.

Early pharmacological studies targeting P2X7 employed antagonists such as Brilliant Blue G. In J20 mice, P2X7 inhibition reduced glycogen synthase kinase-3β (GSK-3β) activity, promoted the α-secretase pathway, and decreased hippocampal Aβ plaque burden; however, these early compounds were limited by insufficient selectivity and brain exposure (Diaz-Hernandez et al., 2012). The brain-penetrant negative allosteric modulator UB-ALT-P2, reported in 2026, represents a recent advance in this field. This compound exhibits oral bioavailability and a prolonged binding residence time at P2X7. In 5xFAD mice, UB-ALT-P2 improved novel object recognition memory and reduced Aβ plaque burden, AT8-reactive phosphorylated tau (p-tau), oxidative stress, and inflammatory markers (Turcu et al., 2026). However, this study has not yet undergone peer review and requires independent replication and systematic toxicological evaluation; therefore, its findings should not be regarded as evidence of clinical efficacy.

Compared with upstream P2X7 blockade, direct inhibition of NLRP3 may intercept a broader range of danger signals. In APP/PS1 mice, MCC950 inhibited caspase-1 activation and IL-1β release while restoring non-phlogistic microglial phagocytosis of Aβ, thereby reducing Aβ burden and improving cognitive function (Dempsey et al., 2017). Oral administration of the NLRP3 inhibitor dapansutrile (OLT1177) to 6-month-old APP/PS1 mice for 3 months improved spatial learning, synaptic plasticity, and microglial responses and reduced cortical plaque burden (Lonnemann et al., 2020). In addition, the selective, blood–brain barrier-penetrant investigational compound VEN-02XX was administered orally for 9 consecutive weeks to symptomatic 5xFAD/Rubicon-knockout mice. Treatment dose-dependently improved learning and memory and reduced reactive microgliosis, inflammatory mediators, phosphorylated tau, and plasma neurofilament light chain levels. VEN-02XX also reduced plaque size, although the reduction in plaque number did not reach statistical significance (Auger et al., 2025). This post-symptomatic intervention paradigm more closely resembles the clinical setting than preventive treatment; however, because Rubicon deficiency accelerates inflammation and pathological progression, these findings require validation in additional AD models.

The cGAS–STING pathway primarily detects double-stranded DNA that is aberrantly localized in the cytosol. Aβ and pathological tau can induce mitochondrial stress in microglia and increase the cytosolic accumulation of ectopic DNA, including mitochondrial DNA (mtDNA). Binding of cytosolic DNA activates cGAS, which catalyzes the synthesis of 2′,3′-cGAMP and subsequently activates STING. Activated STING induces the expression of IFN-β, CXCL10, and other inflammatory mediators through the TBK1–IRF3 and NF-κB signaling axes, thereby sustaining type I interferon and neuroinflammatory responses (Sun et al., 2013; Zhang et al., 2013; Udeochu et al., 2023; Chung et al., 2024). Genetic deletion of *Cgas* attenuated Aβ deposition, gliosis, and cognitive impairment in 5xFAD mice, and the STING inhibitor H-151 produced similar protective effects (Xie et al., 2023). In P301S tau mice, the brain-penetrant cGAS inhibitor TDI-6570 suppressed the interferon response and restored the neuronal MEF2C transcriptional network, synaptic density, long-term potentiation, and memory function. The human cGAS inhibitor TDI-8246 also reduced CXCL10 and CCL5 expression in tau-stimulated human induced pluripotent stem cell-derived microglia (Udeochu et al., 2023). Further studies showed that treatment of *App^NL–G–F^*/hTau double-knock-in mice with H-151 for 2.5 months simultaneously reduced Aβ burden, tau phosphorylation, microglial engulfment of synapses, and memory impairment. This study also demonstrated that STING activity was required for NLRP3 activation in microglia, suggesting that the cGAS–STING and NLRP3 pathways do not operate independently but may converge during inflammatory amplification (Chung et al., 2024). Inducible, microglia-specific deletion of Cgas in 5xFAD mice further limited plaque accumulation, inflammasome activation, and cognitive impairment, providing causal evidence for cell type-specific intervention (He et al., 2025).

Overall, P2X7 blockade may be particularly suited to suppressing ATP-driven upstream danger signaling, whereas direct NLRP3 inhibition can block IL-1β/ASC-mediated amplification downstream of multiple convergent stimuli. Inhibition of cGAS or STING may additionally modulate type I interferon signaling and NLRP3-associated responses. However, these pathways also contribute to host defense, cellular clearance, and tissue repair; prolonged and complete inhibition could therefore compromise immune surveillance within the brain. Moreover, findings are not entirely consistent across experimental models. For example, deficiency of either *Nlrp3* or microglial *Gsdmd* did not ameliorate the highly aggressive P301S tauopathy phenotype (Paesmans et al., 2024). Future translational efforts should therefore focus on developing inhibitors with blood–brain barrier permeability, cell type selectivity, and tunable pharmacodynamic duration rather than indiscriminately suppressing inflammation. Patient selection should incorporate Aβ and tau burden, ASC–IL-1β activity, and interferon signatures. At present, evidence supporting the efficacy of these agents in AD remains largely restricted to cellular and murine models. Further preclinical development must address species-specific differences, the optimal therapeutic window, and the safety risks associated with chronic immunosuppression.

### 4.3 Therapeutic strategies targeting microglial survival and synaptic engulfment: CSF1R and the complement pathway

CSF1R and the complement pathway regulate microglial population size and the selection of phagocytic targets, respectively. From a therapeutic perspective, determining whether the expansion of pathogenic microglia can be restricted while preserving their functions in plaque containment, cellular debris clearance, and synaptic maintenance may have greater translational relevance. Both CSF-1 and IL-34 bind to and activate CSF1R, inducing receptor dimerization and autophosphorylation and subsequently initiating downstream signaling networks, including the PI3K–AKT and RAS–RAF–MEK–ERK pathways. CSF1R-dependent signaling is essential for microglial survival and also promotes microglial proliferation (Elmore et al., 2014; Stanley and Chitu, 2014; Hagan et al., 2020; Berglund et al., 2024). Brain-penetrant CSF1R inhibitors can markedly reduce microglial numbers, whereas residual cells expand and repopulate the niche following treatment withdrawal. Thus, CSF1R inhibition is not simply “anti-inflammatory” in the conventional sense but instead resets the size and composition of the microglial population (Elmore et al., 2014). In APP/PS1 mice, GW2580 inhibited the proliferation of microglia surrounding plaques and improved synaptic integrity and memory without reducing the number of Aβ plaques (Olmos-Alonso et al., 2016). The selective inhibitor JNJ-40346527 (edicotinib) attenuated tau-associated neurodegeneration and functional impairment in the P301S model, suggesting that the benefits of CSF1R inhibition can be dissociated from Aβ clearance (Mancuso et al., 2019). An *ex vivo* study further showed that short-term PLX3397 treatment shifted microglia toward a phagocytic phenotype, promoted Aβ clearance from glutamatergic terminals, and restored long-term potentiation (LTP); however, findings from acute brain slices do not establish chronic efficacy *in vivo* (Piccioni et al., 2024).

The critical determinants of the success of CSF1R-targeted strategies may be disease stage and the degree of inhibition. When 5xFAD mice received PLX5622 before plaque formation, parenchymal plaques were markedly reduced, but Aβ deposition was redirected toward cerebral vessels (Spangenberg et al., 2019). Studies combining genetic and pharmacological depletion further demonstrated that eliminating microglia before plaque formation reduced plaque initiation and neuritic dystrophy. In contrast, microglial depletion after plaque formation decreased plaque compaction and aggravated surrounding neuritic damage, indicating that plaque-associated microglia exert a barrier function during later disease stages (Baligács et al., 2024). Consistent with these findings, PLX3397-induced partial depletion followed by repopulation produced only a transient reduction in plaque burden. Moreover, late-stage plaque-associated microglia were relatively resistant to CSF1R inhibition because of reduced CSF1R expression, potentially resulting in the preferential depletion of homeostatic microglia (Le et al., 2024). These findings help explain the coexistence of beneficial and detrimental effects across different models and indicate that near-complete, prolonged microglial depletion cannot be directly extrapolated to patients. Future development should therefore focus on CNS-selective, partial, or intermittent (pulsed) CSF1R inhibition. CSF1R target occupancy, biomarkers of glial responses, and the risk of cerebral amyloid angiopathy should be integrated to optimize dosing and define appropriate therapeutic windows.

Under conditions of Aβ exposure or certain neurodegenerative insults, C1q can accumulate on vulnerable synapses and initiate the classical complement cascade, generating C3 cleavage fragments, including C3b and iC3b (Stevens et al., 2007; Hong et al., 2016; Carpanini et al., 2022). Synapses opsonized by these complement fragments can be recognized by complement receptor 3 (CR3) on microglia and engulfed through the C3–CR3 axis (Schafer et al., 2012; Hong et al., 2016; Fernández et al., 2022). Meanwhile, C3a and C5a generated during complement activation can promote microglial and astrocytic reactivity through C3aR and C5aR1, respectively, thereby amplifying neuroinflammation at specific disease stages (Lian et al., 2016; Litvinchuk et al., 2018; Schartz et al., 2024). In mouse models of AD, the C1q–C3–CR3 axis contributes to synapse loss before overt plaque formation (Hong et al., 2016). A C1q-blocking antibody inhibited microglial synapse removal and restored synaptic density in a P301S tauopathy model (Dejanovic et al., 2018). A subsequent study showed that conditional deletion of C1q specifically in microglia in young adult mice at 8 weeks of age reduced synaptic engulfment and improved spatial memory without altering overall Aβ burden. However, plaque compaction was reduced, indicating that even cell-specific interventions require monitoring of plaque morphology and vascular risk (Petrisko et al., 2026). The humanized anti-C1q antibody ANX005 has achieved C1q inhibition in both serum and cerebrospinal fluid in patients with Guillain–Barré syndrome; however, this finding demonstrates only pharmacological feasibility in humans and cannot substitute for validation of efficacy in AD (Mohammad et al., 2025). In addition, C1q contributes to the clearance of apoptotic cells and tissue debris, and the infection risks and homeostatic consequences of long-term systemic blockade remain to be evaluated.

Downstream of the complement cascade, selective inhibition of C5aR1 may preserve more opsonization-mediated clearance and tissue-repair functions than prolonged blockade of C1q or C3. In the Arctic48 and Tg2576 models, PMX205 reduced the loss of VGLUT1-positive synapses and improved LTP in a brain region- and age-dependent manner (Gomez-Arboledas et al., 2024). Single-cell studies further showed that PMX205 suppressed detrimental transcriptional programs in microglia and astrocytes and preserved short-term memory in female Arctic48 mice without altering hippocampal plaque burden (Schartz et al., 2024). Nevertheless, these findings remain confined to animal models; earlier treatment was more pronounced in affecting biomarkers of neuronal injury, and there is currently no evidence of efficacy in patients with AD. Therefore, both CSF1R and the complement pathway should be regarded as stage-dependent targets with restricted therapeutic windows rather than universally beneficial anti-inflammatory targets.

### 4.4 Selective modulation of TNF signaling and RIPK1-mediated cell-fate determination

A more mechanistically grounded approach is to distinguish potentially pathogenic inflammatory amplification from physiological immunoregulatory functions along the TNF–TNFR1–RIPK1 signaling axis, thereby reducing potential interference with immune homeostasis and tissue repair. Tumor necrosis factor (TNF) is expressed on the cell membrane in a transmembrane form (tmTNF) and can subsequently be cleaved by ADAM17 to generate soluble TNF (sTNF). These two forms differ in their receptor preferences and signaling characteristics (Black et al., 1997; Moss et al., 1997). TNFR1 is widely expressed, whereas TNFR2 is enriched in immunoregulatory cell populations; accordingly, the two TNF receptors exhibit a degree of functional divergence at the receptor level (Wajant and Siegmund, 2019). sTNF predominantly activates TNFR1, initiating proinflammatory NF-κB/MAPK signaling and enhancing microglial responses. When cell-survival checkpoints are compromised, it can also induce apoptosis or necroptosis through RIPK1-related mechanisms (Su et al., 2022; Huyghe et al., 2023; Preedy et al., 2024; Zhang et al., 2024). By contrast, tmTNF more efficiently activates TNFR2 and promotes cell survival, tissue repair, and immune homeostasis through pathways including NF-κB and PI3K–AKT, while also supporting the expansion and suppressive function of regulatory T cells (Medler et al., 2022; Williams et al., 2024; Tucci et al., 2025).

Conventional TNF antagonists, including etanercept and adalimumab, bind both sTNF and tmTNF, although their effects on tmTNF-expressing cells are not identical (Kaymakcalan et al., 2009; Horiuchi et al., 2010). Both agents are systemic immunomodulators, lack microglial specificity, and exhibit limited penetration across the blood–brain barrier (Chang et al., 2017). In a Phase II randomized controlled trial involving 41 patients with mild-to-moderate AD, 24 weeks of subcutaneous etanercept treatment primarily demonstrated short-term tolerability, with no significant improvements in cognition, behavior, or global function (Butchart et al., 2015). No randomized controlled trial demonstrating the efficacy of adalimumab in AD has been published, and the existing evidence from animal experiments, case reports, and retrospective association studies cannot establish that adalimumab improves clinical outcomes in patients with AD (Camargo et al., 2015; Park et al., 2019; Zhou et al., 2020). XPro1595 is a dominant-negative TNF variant that forms inactive heterotrimers with endogenous sTNF, thereby selectively inhibiting sTNF while largely preserving tmTNF signaling. However, its selectivity is directed toward the molecular form of TNF rather than toward microglia. In 5xFAD mice, XPro1595 altered brain immune-cell profiles, reduced Aβ plaque burden, and improved synaptic plasticity; however, findings from animal models cannot be directly extrapolated to human AD (MacPherson et al., 2017).

RIPK1 functions downstream of TNFR1 and other inflammatory receptors as a molecular switch coordinating inflammatory transcription, cell survival, and programmed cell death. Within TNFR1 complex I, RIPK1 primarily performs a kinase-independent scaffolding function, and its ubiquitination facilitates the recruitment of the TAK1 and IKK complexes, thereby activating NF-κB/MAPK signaling and suppressing cell death. When RIPK1 ubiquitination or checkpoints such as TAK1–IKK are impaired, RIPK1 kinase activity is activated, promoting the formation of death-inducing complexes. Caspase-8 activation can mediate RIPK1-dependent apoptosis, whereas inhibition of caspase-8 activity permits necroptosis through the RIPK3–MLKL axis (Annibaldi et al., 2018; Newton et al., 2019).

Accordingly, small-molecule inhibitors primarily target RIPK1 kinase activity and may theoretically limit pathological inflammation and cell death while preserving part of its scaffolding function (Mifflin et al., 2020). Increased RIPK1 has been observed in plaque-associated microglia in APP/PS1 mice and human AD brain tissue. Genetic or pharmacological inhibition of RIPK1 attenuated aberrant microglial transcriptional programs, lysosomal dysfunction, and Aβ burden; however, this evidence remains predominantly derived from amyloid-based mouse models (Ofengeim et al., 2017). Phase I/Ib studies of the CNS-penetrant RIPK1 inhibitor SAR443060 (DNL747) further illustrate the limitations of early pharmacological evidence. In 16 patients with AD, administration of 50 mg twice daily for 28 days resulted in cerebrospinal fluid exposure and an 81.83% reduction in pRIPK1 in peripheral blood mononuclear cells at the steady-state trough concentration. However, these findings demonstrate only CNS exposure and peripheral target engagement and do not establish adequate RIPK1 inhibition in microglia. The study also detected no significant change in the exploratory digital clock-drawing measure and lacked sufficient statistical power to evaluate clinical efficacy (Vissers et al., 2022). More importantly, development of SAR443060 was discontinued due to long-term nonclinical toxicology findings. Because comparable toxicity has not been reproduced with other RIPK1 inhibitors, these findings may be compound-specific and should not simply be generalized as a class effect of RIPK1 inhibition. The subsequent compound SAR443820 (DNL788) has demonstrated cerebrospinal fluid exposure and peripheral target engagement in healthy participants but has not provided evidence of efficacy in AD (Hincelin-Mery et al., 2024).

Overall, studies of sTNF and RIPK1 support a transition in AD anti-inflammatory therapy from nonselective immunosuppression toward interventions guided by ligand form, signaling node, and biomarkers. Nevertheless, the available evidence remains insufficient to establish clinical efficacy. Future trials will need to establish central pharmacodynamic effects, incorporate patient-enrichment biomarkers directly linked to the therapeutic target, employ clinically meaningful cognitive and functional endpoints, and conduct sufficiently long-term toxicological and safety assessments to complete the evidentiary chain from mechanistic plausibility to disease-modifying efficacy.

## 5 Discussion

This review integrates recent evidence from genetic, single-cell/single-nucleus, and spatial transcriptomic studies with research on Aβ plaque remodeling, tau propagation, synaptic injury, and therapeutic translation within a unified “cellular state–pathological network–therapeutic window” framework. Moving beyond the M1/M2 dichotomy and simplistic “pro-inflammatory/anti-inflammatory” narratives, current evidence indicates that microglia occupy a continuum of overlapping, interconvertible, and highly context-dependent functional states, whose effects are jointly shaped by genetic background, the type of pathology, and disease stage. Disease-associated microglia and transcriptional programs related to interferon responses, antigen presentation, and lipid metabolism should therefore not be regarded as AD-specific or functionally fixed cellular subtypes (Keren-Shaul et al., 2017; Sierksma et al., 2020; Chen and Colonna, 2021; Hou et al., 2022; Paolicelli et al., 2022; Singh et al., 2022; Wang S. et al., 2022; Prater et al., 2023; Guvenek et al., 2024; Mancuso et al., 2024; Martins-Ferreira et al., 2025). Before plaque formation, microglia may participate in Aβ seeding and local deposition; after plaque formation, they may promote plaque compaction and limit damage to surrounding neurites (d’Errico et al., 2022; Baligács et al., 2024). Microglial uptake of extracellular tau may initially facilitate its clearance. However, following persistent exposure to aggregated tau, signaling pathways involving NF-κB, NLRP3–ASC, and PQBP1–cGAS–STING can amplify inflammatory responses, promote the release of tau seeds, and propagate tau pathology. In turn, inflammatory mediators such as IL-1β and TNF-α can exacerbate abnormal tau phosphorylation and neuronal stress (Kitazawa et al., 2011; Ising et al., 2019; Stancu et al., 2019; Malpetti et al., 2020; Jin et al., 2021; Ou et al., 2021; Wang C. et al., 2022; Chen and Yu, 2023). Thus, Aβ, tau, cellular injury, and neuroinflammation are better understood as a mutually coupled feedback network rather than a linear cascade initiated solely by Aβ.

Available clinical findings indicate that mechanistic plausibility and target engagement do not necessarily translate into disease-modifying effects. Although the TREM2 agonistic antibody AL002 demonstrated central nervous system target engagement in the phase 2 INVOKE-2 trial, it did not meet its primary efficacy endpoint (Mummery et al., 2026). Similarly, no clinical evidence currently supports the efficacy of the CD33 antibody AL003 (Hampel et al., 2020). A small randomized controlled trial of etanercept primarily established short-term tolerability and did not demonstrate significant improvements in cognition, behavior, or global function (Butchart et al., 2015). Likewise, the cerebrospinal fluid exposure and peripheral target inhibition observed with the RIPK1 inhibitor SAR443060 do not establish adequate RIPK1 modulation within microglia or improvement in clinical outcomes (Vissers et al., 2022). Moreover, deficiency of either NLRP3 or microglial GSDMD failed to ameliorate the aggressive phenotype of the P301S tauopathy model (Paesmans et al., 2024). These findings do not negate the biological relevance of these targets but highlight a critical evidentiary gap between target occupancy, pharmacodynamic changes, and cognitive or functional benefits.

Future studies should prioritize patient stratification, therapeutic timing, and the validation of central pharmacodynamic effects. Patient selection may integrate Aβ and tau burden, *APOE*/*TREM2*/*CD33* genetic backgrounds, and biomarkers of immune activation and neuronal injury. In addition to assessing peripheral inflammatory changes, clinical trials should verify blood–brain barrier penetration, central target occupancy, and microglia-related pharmacodynamic effects while incorporating clinically meaningful cognitive, functional, and imaging endpoints. The longitudinal integration of single-cell and spatial multi-omics, *in vivo* imaging, and fluid biomarkers may help identify the predominant inflammatory pathways in different patients and at different disease stages, thereby providing a basis for the rational combination of microglial modulators with anti-Aβ or anti-tau therapies.

This review also has several limitations. The included studies were highly heterogeneous with respect to species, experimental models, brain regions, disease stages, and outcome measures. Most therapeutic evidence still derives from cell-based systems or rapidly progressing mouse models. Moreover, human brain single-cell studies are largely based on cross-sectional postmortem analyses, and technical workflows may influence the identification of microglial states. Accordingly, the current evidence is better suited to supporting mechanistic hypotheses and informing trial design than to drawing definitive conclusions regarding the clinical efficacy of most targets. Overall, future therapies should neither drive microglia toward a predetermined phenotype nor indiscriminately deplete them or suppress all of their functions. Instead, therapeutic strategies should selectively constrain pathogenic signaling according to disease stage and biomarker profiles, while preserving microglia’s adaptive homeostatic functions.

## References

[B1] Annibaldi A. Wicky John S. Vanden Berghe T. Swatek K. N. Ruan J. Liccardi G. et al. (2018). Ubiquitin-mediated regulation of RIPK1 kinase activity independent of IKK and MK2. *Mol. Cell.* 69 566–580.e5. 10.1016/j.molcel.2018.01.027 29452637 PMC5823975

[B2] Atagi Y. Liu C.-C. Painter M. M. Chen X.-F. Verbeeck C. Zheng H. et al. (2015). Apolipoprotein E is a ligand for triggering receptor expressed on myeloid cells 2 (TREM2). *J. Biol. Chem.* 290 26043–26050. 10.1074/jbc.M115.679043 26374899 PMC4646257

[B3] Auger A. Faidi R. Rickman A. D. Martinez C. P. Fajfer A. Carling J. et al. (2025). Post-symptomatic NLRP3 inhibition rescues cognitive impairment and mitigates amyloid and tau driven neurodegeneration. *NPJ Dement.* 1:3. 10.1038/s44400-025-00011-5 40343261 PMC12055592

[B4] Baligács N. Albertini G. Borrie S. C. Serneels L. Pridans C. Balusu S. et al. (2024). Homeostatic microglia initially seed and activated microglia later reshape amyloid plaques in Alzheimer’s disease. *Nat. Commun.* 15:10634. 10.1038/s41467-024-54779-w 39639016 PMC11621353

[B5] Ballard C. Gauthier S. Corbett A. Brayne C. Aarsland D. Jones E. (2011). Alzheimer’s disease. *Lancet* 377 1019–1031. 10.1016/S0140-6736(10)61349-9 21371747

[B6] Berglund R. Cheng Y. Piket E. Adzemovic M. Z. Zeitelhofer M. Olsson T. et al. (2024). The aging mouse CNS is protected by an autophagy-dependent microglia population promoted by IL-34. *Nat. Commun.* 15:383. 10.1038/s41467-023-44556-6 38195627 PMC10776874

[B7] Black R. A. Rauch C. T. Kozlosky C. J. Peschon J. J. Slack J. L. Wolfson M. F. et al. (1997). A metalloproteinase disintegrin that releases tumour-necrosis factor-alpha from cells. *Nature* 385 729–733. 10.1038/385729a0 9034190

[B8] Boche D. Perry V. H. Nicoll J. A. R. (2013). Review: Activation patterns of microglia and their identification in the human brain. *Neuropathol. Appl. Neurobiol.* 39 3–18. 10.1111/nan.12011 23252647

[B9] Bruttger J. Karram K. Wörtge S. Regen T. Marini F. Hoppmann N. et al. (2015). Genetic cell ablation reveals clusters of local self-renewing microglia in the mammalian central nervous system. *Immunity* 43 92–106. 10.1016/j.immuni.2015.06.012 26163371

[B10] Bucci M. Chiotis K. Nordberg A. Alzheimer’s Disease, and Neuroimaging Initiative. (2021). Alzheimer’s disease profiled by fluid and imaging markers: Tau PET best predicts cognitive decline. *Mol. Psychiatry* 26 5888–5898. 10.1038/s41380-021-01263-2 34593971 PMC8758489

[B11] Butchart J. Brook L. Hopkins V. Teeling J. Püntener U. Culliford D. et al. (2015). Etanercept in Alzheimer disease: A randomized, placebo-controlled, double-blind, phase 2 trial. *Neurology* 84 2161–2168. 10.1212/WNL.0000000000001617 25934853 PMC4451045

[B12] Butterfield D. A. Halliwell B. (2019). Oxidative stress, dysfunctional glucose metabolism and Alzheimer disease. *Nat. Rev. Neurosci.* 20 148–160. 10.1038/s41583-019-0132-6 30737462 PMC9382875

[B13] Cai Y. Liu J. Wang B. Sun M. Yang H. (2022). Microglia in the neuroinflammatory pathogenesis of Alzheimer’s disease and related therapeutic targets. *Front. Immunol.* 13:856376. 10.3389/fimmu.2022.856376 35558075 PMC9086828

[B14] Camargo C. H. F. Justus F. F. Retzlaff G. Blood M. R. Y. Schafranski M. D. (2015). Action of anti-TNF-α drugs on the progression of Alzheimer’s disease: A case report. *Dement. Neuropsychol.* 9 196–200. 10.1590/1980-57642015DN92000015 29213962 PMC5619359

[B15] Carpanini S. M. Torvell M. Bevan R. J. Byrne R. A. J. Daskoulidou N. Saito T. et al. (2022). Terminal complement pathway activation drives synaptic loss in Alzheimer’s disease models. *Acta Neuropathol. Commun.* 10:99. 10.1186/s40478-022-01404-w 35794654 PMC9258209

[B16] Chang R. Yee K.-L. Sumbria R. K. (2017). Tumor necrosis factor α Inhibition for Alzheimer’s disease. *J. Cent. Nerv. Syst. Dis.* 9:1179573517709278. 10.1177/1179573517709278 28579870 PMC5436834

[B17] Chen Y. Colonna M. (2021). Microglia in Alzheimer’s disease at single-cell level. Are there common patterns in humans and mice? *J. Exp. Med.* 218:e20202717. 10.1084/jem.20202717 34292312 PMC8302448

[B18] Chen Y. Yu Y. (2023). Tau and neuroinflammation in Alzheimer’s disease: Interplay mechanisms and clinical translation. *J. Neuroinflammation* 20:165. 10.1186/s12974-023-02853-3 37452321 PMC10349496

[B19] Chung S. Jeong J.-H. Park J.-C. Han J. W. Lee Y. Kim J.-I. et al. (2024). Blockade of STING activation alleviates microglial dysfunction and a broad spectrum of Alzheimer’s disease pathologies. *Exp. Mol. Med.* 56 1936–1951. 10.1038/s12276-024-01295-y 39218977 PMC11447230

[B20] Crehan H. Holton P. Wray S. Pocock J. Guerreiro R. Hardy J. (2012). Complement receptor 1 (CR1) and Alzheimer’s disease. *Immunobiology* 217 244–250. 10.1016/j.imbio.2011.07.017 21840620

[B21] d’Errico P. Ziegler-Waldkirch S. Aires V. Hoffmann P. Mezö C. Erny D. et al. (2022). Microglia contribute to the propagation of Aβ into unaffected brain tissue. *Nat Neurosci* 25 20–25. 10.1038/s41593-021-00951-0 34811521 PMC8737330

[B22] de Waard D. M. Bugiani M. (2020). Astrocyte-oligodendrocyte-microglia crosstalk in astrocytopathies. *Front. Cell. Neurosci.* 14:608073. 10.3389/fncel.2020.608073 33328899 PMC7710860

[B23] Dejanovic B. Huntley M. A. De Mazière A. Meilandt W. J. Wu T. Srinivasan K. et al. (2018). Changes in the synaptic proteome in tauopathy and rescue of tau-induced synapse loss by C1q antibodies. *Neuron* 100 1322–1336.e7. 10.1016/j.neuron.2018.10.014 30392797

[B24] Dempsey C. Rubio Araiz A. Bryson K. J. Finucane O. Larkin C. Mills E. L. et al. (2017). Inhibiting the NLRP3 inflammasome with MCC950 promotes non-phlogistic clearance of amyloid-β and cognitive function in APP/PS1 mice. *Brain Behav. Immun.* 61 306–316. 10.1016/j.bbi.2016.12.014 28003153

[B25] Diaz-Hernandez J. I. Gomez-Villafuertes R. León-Otegui M. Hontecillas-Prieto L. Del Puerto A. Trejo J. L. et al. (2012). In vivo P2X7 inhibition reduces amyloid plaques in Alzheimer’s disease through GSK3β and secretases. *Neurobiol. Aging* 33 1816–1828. 10.1016/j.neurobiolaging.2011.09.040 22048123

[B26] Dodd R. B. Enomoto M. Zhou Y. Satoh K. Zhang Y. Chen F. et al. (2026). CD33 and clusterin interact biophysically and genetically to modulate Alzheimer risk. *Nat Commun* 10.1038/s41467-026-75140-3 Online ahead of print. 42469217 PMC13507242

[B27] Doens D. Fernández P. L. (2014). Microglia receptors and their implications in the response to amyloid β for Alzheimer’s disease pathogenesis. *J. Neuroinflammation* 11:48. 10.1186/1742-2094-11-48 24625061 PMC3975152

[B28] Easley-Neal C. Foreman O. Sharma N. Zarrin A. A. Weimer R. M. (2019). CSF1R ligands IL-34 and CSF1 are differentially required for microglia development and maintenance in white and gray matter brain regions. *Front. Immunol.* 10:2199. 10.3389/fimmu.2019.02199 31616414 PMC6764286

[B29] Egan M. F. Kost J. Tariot P. N. Aisen P. S. Cummings J. L. Vellas B. et al. (2018). Randomized trial of verubecestat for mild-to-moderate Alzheimer’s disease. *N. Engl. J. Med.* 378 1691–1703. 10.1056/NEJMoa1706441 29719179 PMC6776074

[B30] Elmore M. R. P. Najafi A. R. Koike M. A. Dagher N. N. Spangenberg E. E. Rice R. A. et al. (2014). Colony-stimulating factor 1 receptor signaling is necessary for microglia viability, unmasking a microglia progenitor cell in the adult brain. *Neuron* 82 380–397. 10.1016/j.neuron.2014.02.040 24742461 PMC4161285

[B31] Eskandari-Sedighi G. Crichton M. Zia S. Gomez-Cardona E. Cortez L. M. Patel Z. H. et al. (2024). Alzheimer’s disease associated isoforms of human CD33 distinctively modulate microglial cell responses in 5XFAD mice. *Mol. Neurodegener.* 19:42. 10.1186/s13024-024-00734-8 38802940 PMC11129479

[B32] Eskandari-Sedighi G. Jung J. Macauley M. S. (2023). CD33 isoforms in microglia and Alzheimer’s disease: Friend and foe. *Mol. Aspects Med.* 90:101111. 10.1016/j.mam.2022.101111 35940942

[B33] Fang F. Lue L.-F. Yan S. Xu H. Luddy J. S. Chen D. et al. (2010). RAGE-dependent signaling in microglia contributes to neuroinflammation, Abeta accumulation, and impaired learning/memory in a mouse model of Alzheimer’s disease. *FASEB J.* 24 1043–1055. 10.1096/fj.09-139634 19906677 PMC3231946

[B34] Fernández F. J. Santos-López J. Martínez-Barricarte R. Querol-García J. Martín-Merinero H. Navas-Yuste S. et al. (2022). The crystal structure of iC3b-CR3 αI reveals a modular recognition of the main opsonin iC3b by the CR3 integrin receptor. *Nat. Commun.* 13:1955. 10.1038/s41467-022-29580-2 35413960 PMC9005620

[B35] Galimberti D. Scarpini E. (2012). Progress in Alzheimer’s disease. *J. Neurol.* 259 201–211. 10.1007/s00415-011-6145-3 21706152

[B36] Geldmacher D. S. (2024). Treatment of Alzheimer disease. *Continuum* 30 1823–1844. 10.1212/CON.0000000000001503 39620846

[B37] Gerrits E. Brouwer N. Kooistra S. M. Woodbury M. E. Vermeiren Y. Lambourne M. et al. (2021). Distinct amyloid-β and tau-associated microglia profiles in Alzheimer’s disease. *Acta Neuropathol.* 141 681–696. 10.1007/s00401-021-02263-w 33609158 PMC8043951

[B38] Gomez-Arboledas A. Fonseca M. I. Kramar E. Chu S.-H. Schartz N. D. Selvan P. et al. (2024). C5aR1 signaling promotes region- and age-dependent synaptic pruning in models of Alzheimer’s disease. *Alzheimers Dement.* 20 2173–2190. 10.1002/alz.13682 38278523 PMC10984438

[B39] Griciuc A. Federico A. N. Natasan J. Forte A. M. McGinty D. Nguyen H. et al. (2020). Gene therapy for Alzheimer’s disease targeting CD33 reduces amyloid beta accumulation and neuroinflammation. *Hum. Mol. Genet.* 29 2920–2935. 10.1093/hmg/ddaa179 32803224 PMC7566501

[B40] Griciuc A. Patel S. Federico A. N. Choi S. H. Innes B. J. Oram M. K. et al. (2019). TREM2 acts downstream of CD33 in modulating microglial pathology in Alzheimer’s disease. *Neuron* 103 820–835.e7. 10.1016/j.neuron.2019.06.010 31301936 PMC6728215

[B41] Griciuc A. Serrano-Pozo A. Parrado A. R. Lesinski A. N. Asselin C. N. Mullin K. et al. (2013). Alzheimer’s disease risk gene CD33 inhibits microglial uptake of amyloid beta. *Neuron* 78 631–643. 10.1016/j.neuron.2013.04.014 23623698 PMC3706457

[B42] Guerreiro R. Wojtas A. Bras J. Carrasquillo M. Rogaeva E. Majounie E. et al. (2013). TREM2 variants in Alzheimer’s disease. *N. Engl. J. Med.* 368 117–127. 10.1056/NEJMoa1211851 23150934 PMC3631573

[B43] Guvenek A. Parikshak N. Zamolodchikov D. Gelfman S. Moscati A. Dobbyn L. et al. (2024). Transcriptional profiling in microglia across physiological and pathological states identifies a transcriptional module associated with neurodegeneration. *Commun. Biol.* 7:1168. 10.1038/s42003-024-06684-7 39294270 PMC11411103

[B44] Hagan N. Kane J. L. Grover D. Woodworth L. Madore C. Saleh J. et al. (2020). CSF1R signaling is a regulator of pathogenesis in progressive MS. *Cell. Death Dis.* 11:904. 10.1038/s41419-020-03084-7 33097690 PMC7584629

[B45] Hampel H. Caraci F. Cuello A. C. Caruso G. Nisticò R. Corbo M. et al. (2020). A path toward precision medicine for neuroinflammatory mechanisms in Alzheimer’s disease. *Front. Immunol.* 11:456. 10.3389/fimmu.2020.00456 32296418 PMC7137904

[B46] Hampel H. Mesulam M.-M. Cuello A. C. Farlow M. R. Giacobini E. Grossberg G. T. et al. (2018). The cholinergic system in the pathophysiology and treatment of Alzheimer’s disease. *Brain* 141 1917–1933. 10.1093/brain/awy132 29850777 PMC6022632

[B47] Hansen D. V. Hanson J. E. Sheng M. (2018). Microglia in Alzheimer’s disease. *J. Cell. Biol.* 217 459–472. 10.1083/jcb.201709069 29196460 PMC5800817

[B48] Harry G. J. (2013). Microglia during development and aging. *Pharmacol. Ther.* 139 313–326. 10.1016/j.pharmthera.2013.04.013 23644076 PMC3737416

[B49] Hatori K. Nagai A. Heisel R. Ryu J. K. Kim S. U. (2002). Fractalkine and fractalkine receptors in human neurons and glial cells. *J. Neurosci. Res.* 69 418–426. 10.1002/jnr.10304 12125082

[B50] He S. Li X. Mittra N. Bhattacharjee A. Wang H. Song S. et al. (2025). Microglial cGAS deletion preserves intercellular communication and alleviates amyloid-β-induced pathogenesis of Alzheimer’s disease. *Adv. Sci.* 12:e2410910. 10.1002/advs.202410910 39908354 PMC11948024

[B51] Heneka M. T. Carson M. J. El Khoury J. Landreth G. E. Brosseron F. Feinstein D. L. et al. (2015). Neuroinflammation in Alzheimer’s disease. *Lancet Neurol.* 14 388–405. 10.1016/S1474-4422(15)70016-5 25792098 PMC5909703

[B52] Heneka M. T. Kummer M. P. Stutz A. Delekate A. Schwartz S. Vieira-Saecker A. et al. (2013). NLRP3 is activated in Alzheimer’s disease and contributes to pathology in APP/PS1 mice. *Nature* 493 674–678. 10.1038/nature11729 23254930 PMC3812809

[B53] Hickman S. E. Kingery N. D. Ohsumi T. K. Borowsky M. L. Wang L. Means T. K. et al. (2013). The microglial sensome revealed by direct RNA sequencing. *Nat. Neurosci.* 16 1896–1905. 10.1038/nn.3554 24162652 PMC3840123

[B54] Hincelin-Mery A. Nicolas X. Cantalloube C. Pomponio R. Lewanczyk P. Benamor M. et al. (2024). Safety, pharmacokinetics, and target engagement of a brain penetrant RIPK1 inhibitor, SAR443820 (DNL788), in healthy adult participants. *Clin. Transl. Sci.* 17:e13690. 10.1111/cts.13690 38010108 PMC10772668

[B55] Holers V. M. (2014). Complement and its receptors: New insights into human disease. *Annu. Rev. Immunol.* 32 433–459. 10.1146/annurev-immunol-032713-120154 24499275

[B56] Hong S. Beja-Glasser V. F. Nfonoyim B. M. Frouin A. Li S. Ramakrishnan S. et al. (2016). Complement and microglia mediate early synapse loss in Alzheimer mouse models. *Science* 352 712–716. 10.1126/science.aad8373 27033548 PMC5094372

[B57] Horiuchi T. Mitoma H. Harashima S. Tsukamoto H. Shimoda T. (2010). Transmembrane TNF-alpha: Structure, function and interaction with anti-TNF agents. *Rheumatology* 49 1215–1228. 10.1093/rheumatology/keq031 20194223 PMC2886310

[B58] Hou J. Chen Y. Grajales-Reyes G. Colonna M. (2022). TREM2 dependent and independent functions of microglia in Alzheimer’s disease. *Mol. Neurodegener.* 17:84. 10.1186/s13024-022-00588-y 36564824 PMC9783481

[B59] Huang Y. Xu W. Zhou R. (2021). NLRP3 inflammasome activation and cell death. *Cell. Mol. Immunol.* 18 2114–2127. 10.1038/s41423-021-00740-6 34321623 PMC8429580

[B60] Husemann J. Loike J. D. Anankov R. Febbraio M. Silverstein S. C. (2002). Scavenger receptors in neurobiology and neuropathology: Their role on microglia and other cells of the nervous system. *Glia* 40 195–205. 10.1002/glia.10148 12379907

[B61] Huyghe J. Priem D. Bertrand M. J. M. (2023). Cell death checkpoints in the TNF pathway. *Trends Immunol.* 44 628–643. 10.1016/j.it.2023.05.007 37357102

[B62] Ising C. Venegas C. Zhang S. Scheiblich H. Schmidt S. V. Vieira-Saecker A. et al. (2019). NLRP3 inflammasome activation drives tau pathology. *Nature* 575 669–673. 10.1038/s41586-019-1769-z 31748742 PMC7324015

[B63] Jin M. Shiwaku H. Tanaka H. Obita T. Ohuchi S. Yoshioka Y. et al. (2021). Tau activates microglia via the PQBP1-cGAS-STING pathway to promote brain inflammation. *Nat. Commun.* 12:6565. 10.1038/s41467-021-26851-2 34782623 PMC8592984

[B64] Karran E. De Strooper B. (2022). The amyloid hypothesis in Alzheimer disease: New insights from new therapeutics. *Nat. Rev. Drug Discov.* 21 306–318. 10.1038/s41573-022-00391-w 35177833

[B65] Kaymakcalan Z. Sakorafas P. Bose S. Scesney S. Xiong L. Hanzatian D. K. et al. (2009). Comparisons of affinities, avidities, and complement activation of adalimumab, infliximab, and etanercept in binding to soluble and membrane tumor necrosis factor. *Clin. Immunol.* 131 308–316. 10.1016/j.clim.2009.01.002 19188093

[B66] Keren-Shaul H. Spinrad A. Weiner A. Matcovitch-Natan O. Dvir-Szternfeld R. Ulland T. K. et al. (2017). A Unique microglia type associated with restricting development of Alzheimer’s disease. *Cell* 169 1276–1290.e17. 10.1016/j.cell.2017.05.018 28602351

[B67] Kigerl K. A. de Rivero Vaccari J. P. Dietrich W. D. Popovich P. G. Keane R. W. (2014). Pattern recognition receptors and central nervous system repair. *Exp. Neurol.* 258 5–16. 10.1016/j.expneurol.2014.01.001 25017883 PMC4974939

[B68] Kim S. Y. Moon J. H. Lee H. G. Kim S. U. Lee Y. B. (2007). ATP released from beta-amyloid-stimulated microglia induces reactive oxygen species production in an autocrine fashion. *Exp. Mol. Med.* 39 820–827. 10.1038/emm.2007.89 18160853

[B69] Kirschning C. J. Wesche H. Merrill Ayres T. Rothe M. (1998). Human toll-like receptor 2 confers responsiveness to bacterial lipopolysaccharide. *J. Exp. Med.* 188 2091–2097. 10.1084/jem.188.11.2091 9841923 PMC2212382

[B70] Kitazawa M. Cheng D. Tsukamoto M. R. Koike M. A. Wes P. D. Vasilevko V. et al. (2011). Blocking IL-1 signaling rescues cognition, attenuates tau pathology, and restores neuronal β-catenin pathway function in an Alzheimer’s disease model. *J. Immunol.* 187 6539–6549. 10.4049/jimmunol.1100620 22095718 PMC4072218

[B71] Köhl J. (2006). The role of complement in danger sensing and transmission. *Immunol. Res.* 34 157–176. 10.1385/IR:34:2:157 16760575

[B72] Krasemann S. Madore C. Cialic R. Baufeld C. Calcagno N. El Fatimy R. et al. (2017). The TREM2-APOE pathway drives the transcriptional phenotype of dysfunctional microglia in neurodegenerative diseases. *Immunity* 47 566–581.e9. 10.1016/j.immuni.2017.08.008 28930663 PMC5719893

[B73] La Joie R. Visani A. V. Baker S. L. Brown J. A. Bourakova V. Cha J. et al. (2020). Prospective longitudinal atrophy in Alzheimer’s disease correlates with the intensity and topography of baseline tau-PET. *Sci. Transl. Med.* 12:eaau5732. 10.1126/scitranslmed.aau5732 31894103 PMC7035952

[B74] Lagarde J. Olivieri P. Tonietto M. Tissot C. Rivals I. Gervais P. et al. (2022). Tau-PET imaging predicts cognitive decline and brain atrophy progression in early Alzheimer’s disease. *J. Neurol. Neurosurg. Psychiatry* 93 459–467. 10.1136/jnnp-2021-328623 35228270

[B75] Lannfelt L. Relkin N. R. Siemers E. R. (2014). Amyloid-ß-directed immunotherapy for Alzheimer’s disease. *J. Intern. Med.* 275 284–295. 10.1111/joim.12168 24605809 PMC4238820

[B76] Laumonnier Y. Karsten C. M. Köhl J. (2017). Novel insights into the expression pattern of anaphylatoxin receptors in mice and men. *Mol. Immunol.* 89 44–58. 10.1016/j.molimm.2017.05.019 28600003

[B77] Lawson L. J. Perry V. H. Dri P. Gordon S. (1990). Heterogeneity in the distribution and morphology of microglia in the normal adult mouse brain. *Neuroscience* 39 151–170. 10.1016/0306-4522(90)90229-w 2089275

[B78] Le L. H. D. O’Banion M. K. Majewska A. K. (2024). Partial microglial depletion and repopulation exert subtle but differential effects on amyloid pathology at different disease stages. *Sci. Rep.* 14:30912. 10.1038/s41598-024-81910-0 39730671 PMC11680822

[B79] Lee J. D. Coulthard L. G. Woodruff T. M. (2019). Complement dysregulation in the central nervous system during development and disease. *Semin. Immunol.* 45:101340. 10.1016/j.smim.2019.101340 31708347

[B80] Leng F. Edison P. (2021). Neuroinflammation and microglial activation in Alzheimer disease: Where do we go from here? *Nat. Rev. Neurol.* 17 157–172. 10.1038/s41582-020-00435-y 33318676

[B81] Li T. Lu L. Pember E. Li X. Zhang B. Zhu Z. (2022). New insights into neuroinflammation involved in pathogenic mechanism of Alzheimer’s disease and its potential for therapeutic intervention. *Cells* 11:1925. 10.3390/cells11121925 35741054 PMC9221885

[B82] Li Y. Xia X. Wang Y. Zheng J. C. (2022). Mitochondrial dysfunction in microglia: A novel perspective for pathogenesis of Alzheimer’s disease. *J Neuroinflammation* 19:248. 10.1186/s12974-022-02613-9 36203194 PMC9535890

[B83] Lian H. Litvinchuk A. Chiang A. C.-A. Aithmitti N. Jankowsky J. L. Zheng H. (2016). Astrocyte-microglia cross talk through complement activation modulates amyloid pathology in mouse models of Alzheimer’s disease. *J. Neurosci.* 36 577–589. 10.1523/JNEUROSCI.2117-15.2016 26758846 PMC4710776

[B84] Liao Y. Cheng J. Kong X. Li S. Li X. Zhang M. et al. (2020). HDAC3 inhibition ameliorates ischemia/reperfusion-induced brain injury by regulating the microglial cGAS-STING pathway. *Theranostics* 10 9644–9662. 10.7150/thno.47651 32863951 PMC7449914

[B85] Litvinchuk A. Wan Y.-W. Swartzlander D. B. Chen F. Cole A. Propson N. E. et al. (2018). Complement C3aR inactivation attenuates Tau pathology and reverses an immune network deregulated in tauopathy models and Alzheimer’s disease. *Neuron* 100 1337–1353.e5. 10.1016/j.neuron.2018.10.031 30415998 PMC6309202

[B86] Long J. M. Holtzman D. M. (2019). Alzheimer disease: An update on pathobiology and treatment strategies. *Cell* 179 312–339. 10.1016/j.cell.2019.09.001 31564456 PMC6778042

[B87] Lonnemann N. Hosseini S. Marchetti C. Skouras D. B. Stefanoni D. D’Alessandro A. et al. (2020). The NLRP3 inflammasome inhibitor OLT1177 rescues cognitive impairment in a mouse model of Alzheimer’s disease. *Proc. Natl. Acad. Sci. U S A.* 117 32145–32154. 10.1073/pnas.2009680117 33257576 PMC7749353

[B88] López-González I. Schlüter A. Aso E. Garcia-Esparcia P. Ansoleaga B. LLorens F. et al. (2015). Neuroinflammatory signals in Alzheimer disease and APP/PS1 transgenic mice: Correlations with plaques, tangles, and oligomeric species. *J. Neuropathol. Exp. Neurol.* 74 319–344. 10.1097/NEN.0000000000000176 25756590

[B89] Lu A. Chen W.-T. Dalby M. Sainz Garcia D. Vanheusden M. de Vries L. E. et al. (2026). Human microglial transitions at the Aβ-Tau inflection point associate with divergent pathways to dementia and resilience. *Nat. Med.* 32 2047–2059. 10.1038/s41591-026-04393-8 42243549 PMC13278961

[B90] MacPherson K. P. Sompol P. Kannarkat G. T. Chang J. Sniffen L. Wildner M. E. et al. (2017). Peripheral administration of the soluble TNF inhibitor XPro1595 modifies brain immune cell profiles, decreases beta-amyloid plaque load, and rescues impaired long-term potentiation in 5xFAD mice. *Neurobiol. Dis.* 102 81–95. 10.1016/j.nbd.2017.02.010 28237313 PMC5464789

[B91] Madore C. Joffre C. Delpech J. C. De Smedt-Peyrusse V. Aubert A. Coste L. et al. (2013). Early morphofunctional plasticity of microglia in response to acute lipopolysaccharide. *Brain Behav. Immun.* 34 151–158. 10.1016/j.bbi.2013.08.008 23994463

[B92] Malpetti M. Kievit R. A. Passamonti L. Jones P. S. Tsvetanov K. A. Rittman T. et al. (2020). Microglial activation and tau burden predict cognitive decline in Alzheimer’s disease. *Brain* 143 1588–1602. 10.1093/brain/awaa088 32380523 PMC7241955

[B93] Mancuso R. Fattorelli N. Martinez-Muriana A. Davis E. Wolfs L. Van Den Daele J. et al. (2024). Xenografted human microglia display diverse transcriptomic states in response to Alzheimer’s disease-related amyloid-β pathology. *Nat. Neurosci.* 27 886–900. 10.1038/s41593-024-01600-y 38539015 PMC11089003

[B94] Mancuso R. Fryatt G. Cleal M. Obst J. Pipi E. Monzón-Sandoval J. et al. (2019). CSF1R inhibitor JNJ-40346527 attenuates microglial proliferation and neurodegeneration in P301S mice. *Brain* 142 3243–3264. 10.1093/brain/awz241 31504240 PMC6794948

[B95] Martinon F. Burns K. Tschopp J. (2002). The inflammasome: A molecular platform triggering activation of inflammatory caspases and processing of proIL-beta. *Mol. Cell.* 10 417–426. 10.1016/s1097-2765(02)00599-3 12191486

[B96] Martins-Ferreira R. Calafell-Segura J. Leal B. Rodríguez-Ubreva J. Martínez-Saez E. Mereu E. et al. (2025). The Human Microglia Atlas (HuMicA) unravels changes in disease-associated microglia subsets across neurodegenerative conditions. *Nat. Commun.* 16:739. 10.1038/s41467-025-56124-1 39820004 PMC11739505

[B97] McFarland K. N. Chakrabarty P. (2022). Microglia in Alzheimer’s disease: A key player in the transition between homeostasis and pathogenesis. *Neurotherapeutics* 19 186–208. 10.1007/s13311-021-01179-3 35286658 PMC9130399

[B98] McLarnon J. G. Ryu J. K. Walker D. G. Choi H. B. (2006). Upregulated expression of purinergic P2X(7) receptor in Alzheimer disease and amyloid-beta peptide-treated microglia and in peptide-injected rat hippocampus. *J. Neuropathol. Exp. Neurol.* 65 1090–1097. 10.1097/01.jnen.0000240470.97295.d3 17086106

[B99] Medler J. Kucka K. Wajant H. (2022). Tumor necrosis factor receptor 2 (TNFR2): An emerging target in cancer therapy. *Cancers* 14:2603. 10.3390/cancers14112603 35681583 PMC9179537

[B100] Mifflin L. Ofengeim D. Yuan J. (2020). Receptor-interacting protein kinase 1 (RIPK1) as a therapeutic target. *Nat. Rev. Drug Discov.* 19 553–571. 10.1038/s41573-020-0071-y 32669658 PMC7362612

[B101] Milner M. T. Maddugoda M. Götz J. Burgener S. S. Schroder K. (2021). The NLRP3 inflammasome triggers sterile neuroinflammation and Alzheimer’s disease. *Curr. Opin. Immunol.* 68 116–124. 10.1016/j.coi.2020.10.011 33181351

[B102] Mittelbronn M. Dietz K. Schluesener H. J. Meyermann R. (2001). Local distribution of microglia in the normal adult human central nervous system differs by up to one order of magnitude. *Acta Neuropathol.* 101 249–255. 10.1007/s004010000284 11307625

[B103] Mohammad Q. D. Islam Z. Papri N. Hayat S. Jahan I. Azad K. A. K. et al. (2025). Results from a phase 1 study evaluating the safety, tolerability, pharmacokinetics, pharmacodynamics, and efficacy of ANX005, a C1q inhibitor, in patients with guillain-barré syndrome. *J. Peripher. Nerv. Syst.* 30:e70009. 10.1111/jns.70009 40000167 PMC11886941

[B104] Mosher K. I. Wyss-Coray T. (2014). Microglial dysfunction in brain aging and Alzheimer’s disease. *Biochem. Pharmacol.* 88 594–604. 10.1016/j.bcp.2014.01.008 24445162 PMC3972294

[B105] Moss M. L. Jin S. L. Milla M. E. Bickett D. M. Burkhart W. Carter H. L. et al. (1997). Cloning of a disintegrin metalloproteinase that processes precursor tumour-necrosis factor-alpha. *Nature* 385 733–736. 10.1038/385733a0 9034191

[B106] Mummery C. J. Mayorga A. J. Simmons A. Chow T. W. Burgess B. Nguyen T. et al. (2026). The TREM2 agonistic antibody AL002 in early Alzheimer’s disease: A phase 2 randomized trial. *Nat. Med.* 32 1708–1716. 10.1038/s41591-026-04273-1 41787076

[B107] Muñoz-Planillo R. Kuffa P. Martínez-Colón G. Smith B. L. Rajendiran T. M. Núñez G. (2013). K^+^ efflux is the common trigger of NLRP3 inflammasome activation by bacterial toxins and particulate matter. *Immunity* 38 1142–1153. 10.1016/j.immuni.2013.05.016 23809161 PMC3730833

[B108] Muzio L. Viotti A. Martino G. (2021). Microglia in neuroinflammation and neurodegeneration: From understanding to therapy. *Front. Neurosci.* 15:742065. 10.3389/fnins.2021.742065 34630027 PMC8497816

[B109] Newton K. Wickliffe K. E. Dugger D. L. Maltzman A. Roose-Girma M. Dohse M. et al. (2019). Cleavage of RIPK1 by caspase-8 is crucial for limiting apoptosis and necroptosis. *Nature* 574 428–431. 10.1038/s41586-019-1548-x 31511692

[B110] Nimmerjahn A. Kirchhoff F. Helmchen F. (2005). Resting microglial cells are highly dynamic surveillants of brain parenchyma in vivo. *Science* 308 1314–1318. 10.1126/science.1110647 15831717

[B111] Ofengeim D. Mazzitelli S. Ito Y. DeWitt J. P. Mifflin L. Zou C. et al. (2017). RIPK1 mediates a disease-associated microglial response in Alzheimer’s disease. *Proc. Natl. Acad. Sci. U S A.* 114 E8788–E8797. 10.1073/pnas.1714175114 28904096 PMC5642727

[B112] Olmos-Alonso A. Schetters S. T. T. Sri S. Askew K. Mancuso R. Vargas-Caballero M. et al. (2016). Pharmacological targeting of CSF1R inhibits microglial proliferation and prevents the progression of Alzheimer’s-like pathology. *Brain* 139 891–907. 10.1093/brain/awv379 26747862 PMC4766375

[B113] Ossenkoppele R. Pichet Binette A. Groot C. Smith R. Strandberg O. Palmqvist S. et al. (2022). Amyloid and tau PET-positive cognitively unimpaired individuals are at high risk for future cognitive decline. *Nat. Med.* 28 2381–2387. 10.1038/s41591-022-02049-x 36357681 PMC9671808

[B114] Ou W. Yang J. Simanauskaite J. Choi M. Castellanos D. M. Chang R. et al. (2021). Biologic TNF-α inhibitors reduce microgliosis, neuronal loss, and tau phosphorylation in a transgenic mouse model of tauopathy. *J. Neuroinflammation* 18 312. 10.1186/s12974-021-02332-7 34972522 PMC8719395

[B115] Paesmans I. Van Kolen K. Vandermeeren M. Shih P.-Y. Wuyts D. Boone F. et al. (2024). NLRP3 inflammasome activation and pyroptosis are dispensable for tau pathology. *Front. Aging Neurosci.* 16:1459134. 10.3389/fnagi.2024.1459134 39381137 PMC11458539

[B116] Paolicelli R. C. Sierra A. Stevens B. Tremblay M.-E. Aguzzi A. Ajami B. et al. (2022). Microglia states and nomenclature: A field at its crossroads. *Neuron* 110 3458–3483. 10.1016/j.neuron.2022.10.020 36327895 PMC9999291

[B117] Parakalan R. Jiang B. Nimmi B. Janani M. Jayapal M. Lu J. et al. (2012). Transcriptome analysis of amoeboid and ramified microglia isolated from the corpus callosum of rat brain. *BMC Neurosci.* 13:64. 10.1186/1471-2202-13-64 22697290 PMC3441342

[B118] Paresce D. M. Ghosh R. N. Maxfield F. R. (1996). Microglial cells internalize aggregates of the Alzheimer’s disease amyloid beta-protein via a scavenger receptor. *Neuron* 17 553–565. 10.1016/s0896-6273(00)80187-7 8816718

[B119] Park J. Lee S.-Y. Shon J. Kim K. Lee H. J. Kim K. A. et al. (2019). Adalimumab improves cognitive impairment, exerts neuroprotective effects and attenuates neuroinflammation in an Aβ1-40-injected mouse model of Alzheimer’s disease. *Cytotherapy* 21 671–682. 10.1016/j.jcyt.2019.04.054 31076196

[B120] Parvathenani L. K. Tertyshnikova S. Greco C. R. Roberts S. B. Robertson B. Posmantur R. (2003). P2X7 mediates superoxide production in primary microglia and is up-regulated in a transgenic mouse model of Alzheimer’s disease. *J. Biol. Chem.* 278 13309–13317. 10.1074/jbc.M209478200 12551918

[B121] Pascoal T. A. Benedet A. L. Ashton N. J. Kang M. S. Therriault J. Chamoun M. et al. (2021). Microglial activation and tau propagate jointly across Braak stages. *Nat. Med.* 27 1592–1599. 10.1038/s41591-021-01456-w 34446931

[B122] Pawelec P. Ziemka-Nalecz M. Sypecka J. Zalewska T. (2020). The impact of the CX3CL1/CX3CR1 axis in neurological disorders. *Cells* 9:2277. 10.3390/cells9102277 33065974 PMC7600611

[B123] Petrisko T. J. Chu S.-H. Gomez-Arboledas A. Zhang B. Tenner A. J. (2026). Young adult microglial deletion of C1q reduces engulfment of synapses and partially mitigates cognitive impairment in an aggressive Alzheimer’s disease mouse model. *Glia* 74:e70189. 10.1002/glia.70189 42444329 PMC13366034

[B124] Piccioni G. Maisto N. d’Ettorre A. Strimpakos G. Nisticò R. Triaca V. et al. (2024). Switch to phagocytic microglia by CSFR1 inhibition drives amyloid-beta clearance from glutamatergic terminals rescuing LTP in acute hippocampal slices. *Transl. Psychiatry* 14:338. 10.1038/s41398-024-03019-2 39179543 PMC11344079

[B125] Pinti M. Ferraro D. Nasi M. (2021). Microglia activation: A role for mitochondrial DNA? *Neural Regen. Res.* 16 2393–2394. 10.4103/1673-5374.313034 33907013 PMC8374575

[B126] Prater K. E. Green K. J. Mamde S. Sun W. Cochoit A. Smith C. L. et al. (2023). Human microglia show unique transcriptional changes in Alzheimer’s disease. *Nat. Aging* 3 894–907. 10.1038/s43587-023-00424-y 37248328 PMC10353942

[B127] Preedy M. K. White M. R. H. Tergaonkar V. (2024). Cellular heterogeneity in TNF/TNFR1 signalling: Live cell imaging of cell fate decisions in single cells. *Cell Death Dis.* 15:202. 10.1038/s41419-024-06559-z 38467621 PMC10928192

[B128] Preeti K. Sood A. Fernandes V. (2022). Metabolic regulation of glia and their neuroinflammatory role in Alzheimer’s disease. *Cell. Mol. Neurobiol.* 42 2527–2551. 10.1007/s10571-021-01147-7 34515874 PMC11421648

[B129] Prinz M. Jung S. Priller J. (2019). Microglia biology: One century of evolving concepts. *Cell* 179 292–311. 10.1016/j.cell.2019.08.053 31585077

[B130] Reed-Geaghan E. G. Savage J. C. Hise A. G. Landreth G. E. (2009). CD14 and toll-like receptors 2 and 4 are required for fibrillar Aβ-stimulated microglial activation. *J. Neurosci.* 29 11982–11992. 10.1523/JNEUROSCI.3158-09.2009 19776284 PMC2778845

[B131] Sanz J. M. Chiozzi P. Ferrari D. Colaianna M. Idzko M. Falzoni S. et al. (2009). Activation of microglia by amyloid β requires P2X7 receptor expression. *J. Immunol.* 182 4378–4385. 10.4049/jimmunol.0803612 19299738

[B132] Schafer D. P. Lehrman E. K. Kautzman A. G. Koyama R. Mardinly A. R. Yamasaki R. et al. (2012). Microglia sculpt postnatal neural circuits in an activity and complement-dependent manner. *Neuron* 74 691–705. 10.1016/j.neuron.2012.03.026 22632727 PMC3528177

[B133] Schartz N. D. Liang H. Y. Carvalho K. Chu S.-H. Mendoza-Arvilla A. Petrisko T. J. et al. (2024). C5aR1 antagonism suppresses inflammatory glial responses and alters cellular signaling in an Alzheimer’s disease mouse model. *Nat. Commun.* 15:7028. 10.1038/s41467-024-51163-6 39147742 PMC11327341

[B134] Selkoe D. J. (2011). Alzheimer’s disease. *Cold Spring Harb. Perspect. Biol.* 3:a004457. 10.1101/cshperspect.a004457 21576255 PMC3119915

[B135] Shaikh S. B. Nicholson L. F. (2009). Effects of chronic low dose rotenone treatment on human microglial cells. *Mol. Neurodegener.* 4:55. 10.1186/1750-1326-4-55 20042120 PMC2806357

[B136] Shi J. Zhao Y. Wang K. Shi X. Wang Y. Huang H. et al. (2015). Cleavage of GSDMD by inflammatory caspases determines pyroptotic cell death. *Nature* 526 660–665. 10.1038/nature15514 26375003

[B137] Sierksma A. Lu A. Mancuso R. Fattorelli N. Thrupp N. Salta E. et al. (2020). Novel Alzheimer risk genes determine the microglia response to amyloid-β but not to TAU pathology. *EMBO Mol. Med.* 12:e10606. 10.15252/emmm.201910606 31951107 PMC7059012

[B138] Simpson D. S. A. Oliver P. L. (2020). ROS generation in microglia: Understanding oxidative stress and inflammation in neurodegenerative disease. *Antioxidants* 9:743. 10.3390/antiox9080743 32823544 PMC7463655

[B139] Sims J. R. Zimmer J. A. Evans C. D. Lu M. Ardayfio P. Sparks J. et al. (2023). Donanemab in early symptomatic Alzheimer disease: The TRAILBLAZER-ALZ 2 randomized clinical trial. *JAMA* 330 512–527. 10.1001/jama.2023.13239 37459141 PMC10352931

[B140] Singh N. Benoit M. R. Zhou J. Das B. Davila-Velderrain J. Kellis M. et al. (2022). BACE-1 inhibition facilitates the transition from homeostatic microglia to DAM-1. *Sci. Adv.* 8:eabo1286. 10.1126/sciadv.abo1286 35714196 PMC9205595

[B141] Spangenberg E. Severson P. L. Hohsfield L. A. Crapser J. Zhang J. Burton E. A. et al. (2019). Sustained microglial depletion with CSF1R inhibitor impairs parenchymal plaque development in an Alzheimer’s disease model. *Nat. Commun.* 10:3758. 10.1038/s41467-019-11674-z 31434879 PMC6704256

[B142] Sperling R. A. Donohue M. C. Rissman R. A. Johnson K. A. Rentz D. M. Grill J. D. et al. (2024). Amyloid and Tau prediction of cognitive and functional decline in unimpaired older individuals: Longitudinal data from the A4 and LEARN studies. *J. Prev. Alzheimers Dis.* 11 802–813. 10.14283/jpad.2024.122 39044488 PMC11266444

[B143] Stancu I.-C. Cremers N. Vanrusselt H. Couturier J. Vanoosthuyse A. Kessels S. et al. (2019). Aggregated Tau activates NLRP3-ASC inflammasome exacerbating exogenously seeded and non-exogenously seeded Tau pathology in vivo. *Acta Neuropathol.* 137 599–617. 10.1007/s00401-018-01957-y 30721409 PMC6426830

[B144] Stanley E. R. Chitu V. (2014). CSF-1 receptor signaling in myeloid cells. *Cold Spring Harb. Perspect. Biol.* 6:a021857. 10.1101/cshperspect.a021857 24890514 PMC4031967

[B145] Stephan A. H. Barres B. A. Stevens B. (2012). The complement system: An unexpected role in synaptic pruning during development and disease. *Annu. Rev. Neurosci.* 35 369–389. 10.1146/annurev-neuro-061010-113810 22715882

[B146] Stevens B. Allen N. J. Vazquez L. E. Howell G. R. Christopherson K. S. Nouri N. et al. (2007). The classical complement cascade mediates CNS synapse elimination. *Cell* 131 1164–1178. 10.1016/j.cell.2007.10.036 18083105

[B147] Su Z. Dhusia K. Wu Y. (2022). Understanding the functional role of membrane confinements in TNF-mediated signaling by multiscale simulations. *Commun. Biol.* 5:228. 10.1038/s42003-022-03179-1 35277586 PMC8917213

[B148] Sun L. Wu J. Du F. Chen X. Chen Z. J. (2013). Cyclic GMP-AMP synthase is a cytosolic DNA sensor that activates the type I interferon pathway. *Science* 339 786–791. 10.1126/science.1232458 23258413 PMC3863629

[B149] Sun N. Victor M. B. Park Y. P. Xiong X. Scannail A. N. Leary N. et al. (2023). Human microglial state dynamics in Alzheimer’s disease progression. *Cell* 186 4386–4403.e29. 10.1016/j.cell.2023.08.037 37774678 PMC10644954

[B150] Swanson K. V. Deng M. Ting J. P.-Y. (2019). The NLRP3 inflammasome: Molecular activation and regulation to therapeutics. *Nat. Rev. Immunol.* 19 477–489. 10.1038/s41577-019-0165-0 31036962 PMC7807242

[B151] Takeuchi H. Jin S. Wang J. Zhang G. Kawanokuchi J. Kuno R. et al. (2006). Tumor necrosis factor-alpha induces neurotoxicity via glutamate release from hemichannels of activated microglia in an autocrine manner. *J. Biol. Chem.* 281 21362–21368. 10.1074/jbc.M600504200 16720574

[B152] Tremblay M. -È Stevens B. Sierra A. Wake H. Bessis A. Nimmerjahn A. (2011). The role of microglia in the healthy brain. *J. Neurosci.* 31 16064–16069. 10.1523/JNEUROSCI.4158-11.2011 22072657 PMC6633221

[B153] Tucci G. Pacella I. Pinzon Grimaldos A. Rossi A. Cammarata I. Zagaglioni M. et al. (2025). TNF production or TNFR2 expression characterize distinct states of regulatory T cells that cooperate in Treg expansion in cancer and chronic inflammation. *Eur. J. Immunol.* 55:e70062. 10.1002/eji.70062 40977146 PMC12451257

[B154] Turcu A. L. Oken A. C. Griñán-Ferré C. Durner A. Sierra-Marquez J. Hinz-Kowalik S. et al. (2026). A brain-penetrant P2X7R antagonist mitigates Alzheimer’s disease pathology. *bioRxiv [Preprint]* 10.64898/2026.04.06.716621 41993524 PMC13082025

[B155] Udeochu J. C. Amin S. Huang Y. Fan L. Torres E. R. S. Carling G. K. et al. (2023). Tau activation of microglial cGAS-IFN reduces MEF2C-mediated cognitive resilience. *Nat. Neurosci.* 26 737–750. 10.1038/s41593-023-01315-6 37095396 PMC10166855

[B156] Ulland T. K. Song W. M. Huang S. C.-C. Ulrich J. D. Sergushichev A. Beatty W. L. et al. (2017). TREM2 maintains microglial metabolic fitness in Alzheimer’s disease. *Cell* 170 649–663.e13. 10.1016/j.cell.2017.07.023 28802038 PMC5573224

[B157] Vainchtein I. D. Chin G. Cho F. S. Kelley K. W. Miller J. G. Chien E. C. et al. (2018). Astrocyte-derived interleukin-33 promotes microglial synapse engulfment and neural circuit development. *Science* 359 1269–1273. 10.1126/science.aal3589 29420261 PMC6070131

[B158] van Dyck C. H. Swanson C. J. Aisen P. Bateman R. J. Chen C. Gee M. et al. (2023). Lecanemab in early Alzheimer’s disease. *N. Engl. J. Med.* 388 9–21. 10.1056/NEJMoa2212948 36449413

[B159] Veerhuis R. Nielsen H. M. Tenner A. J. (2011). Complement in the brain. *Mol. Immunol.* 48 1592–1603. 10.1016/j.molimm.2011.04.003 21546088 PMC3142281

[B160] Venegas C. Kumar S. Franklin B. S. Dierkes T. Brinkschulte R. Tejera D. et al. (2017). Microglia-derived ASC specks cross-seed amyloid-β in Alzheimer’s disease. *Nature* 552 355–361. 10.1038/nature25158 29293211

[B161] Vissers M. F. J. M. Heuberger J. A. A. C. Groeneveld G. J. Oude Nijhuis J. De Deyn P. P. Hadi S. et al. (2022). Safety, pharmacokinetics and target engagement of novel RIPK1 inhibitor SAR443060 (DNL747) for neurodegenerative disorders: Randomized, placebo-controlled, double-blind phase I/Ib studies in healthy subjects and patients. *Clin. Transl. Sci.* 15 2010–2023. 10.1111/cts.13317 35649245 PMC9372423

[B162] Vitale I. Pietrocola F. Guilbaud E. Aaronson S. A. Abrams J. M. Adam D. et al. (2023). Apoptotic cell death in disease-current understanding of the NCCD 2023. *Cell. Death Differ.* 30 1097–1154. 10.1038/s41418-023-01153-w 37100955 PMC10130819

[B163] Wajant H. Siegmund D. (2019). TNFR1 and TNFR2 in the control of the life and death balance of macrophages. *Front. Cell. Dev. Biol.* 7:91. 10.3389/fcell.2019.00091 31192209 PMC6548990

[B164] Walker D. G. Whetzel A. M. Serrano G. Sue L. I. Beach T. G. Lue L.-F. (2015). Association of CD33 polymorphism rs3865444 with Alzheimer’s disease pathology and CD33 expression in human cerebral cortex. *Neurobiol. Aging* 36 571–582. 10.1016/j.neurobiolaging.2014.09.023 25448602 PMC4315751

[B165] Wang C. Fan L. Khawaja R. R. Liu B. Zhan L. Kodama L. et al. (2022). Microglial NF-κB drives tau spreading and toxicity in a mouse model of tauopathy. *Nat. Commun.* 13:1969. 10.1038/s41467-022-29552-6 35413950 PMC9005658

[B166] Wang C. Zong S. Cui X. Wang X. Wu S. Wang L. et al. (2023). The effects of microglia-associated neuroinflammation on Alzheimer’s disease. *Front. Immunol.* 14:1117172. 10.3389/fimmu.2023.1117172 36911732 PMC9992739

[B167] Wang H. Shen Y. Chuang H. Chiu C. Ye Y. Zhao L. (2019). Neuroinflammation in Alzheimer’s disease: Microglia, molecular participants and therapeutic choices. *Curr. Alzheimer Res.* 16 659–674. 10.2174/1567205016666190503151648 31580243

[B168] Wang L. Pavlou S. Du X. Bhuckory M. Xu H. Chen M. (2019). Glucose transporter 1 critically controls microglial activation through facilitating glycolysis. *Mol. Neurodegener.* 14:2. 10.1186/s13024-019-0305-9 30634998 PMC6329071

[B169] Wang S. Mustafa M. Yuede C. M. Salazar S. V. Kong P. Long H. et al. (2020). Anti-human TREM2 induces microglia proliferation and reduces pathology in an Alzheimer’s disease model. *J. Exp. Med.* 217:e20200785. 10.1084/jem.20200785 32579671 PMC7478730

[B170] Wang S. Sudan R. Peng V. Zhou Y. Du S. Yuede C. M. et al. (2022). TREM2 drives microglia response to amyloid-β via SYK-dependent and -independent pathways. *Cell* 185 4153–4169.e19. 10.1016/j.cell.2022.09.033 36306735 PMC9625082

[B171] Wang Y. Mandelkow E. (2016). Tau in physiology and pathology. *Nat. Rev. Neurosci.* 17 5–21. 10.1038/nrn.2015.1 26631930

[B172] Wang Y. Cella M. Mallinson K. Ulrich J. D. Young K. L. Robinette M. L. et al. (2015). TREM2 lipid sensing sustains the microglial response in an Alzheimer’s disease model. *Cell* 160 1061–1071. 10.1016/j.cell.2015.01.049 25728668 PMC4477963

[B173] Wang Y. Szretter K. J. Vermi W. Gilfillan S. Rossini C. Cella M. et al. (2012). IL-34 is a tissue-restricted ligand of CSF1R required for the development of Langerhans cells and microglia. *Nat. Immunol.* 13 753–760. 10.1038/ni.2360 22729249 PMC3941469

[B174] Williams R. O. Clanchy F. I. Huang Y.-S. Tseng W.-Y. Stone T. W. (2024). TNFR2 signalling in inflammatory diseases. *Best Pract. Res. Clin. Rheumatol.* 38:101941. 10.1016/j.berh.2024.101941 38538489

[B175] Wong E. Malviya M. Jain T. Liao G. P. Kehs Z. Chang J. C. et al. (2024). HuM195 and its single-chain variable fragment increase Aβ phagocytosis in microglia via elimination of CD33 inhibitory signaling. *Mol. Psychiatry* 29 2084–2094. 10.1038/s41380-024-02474-z 38383769 PMC11336028

[B176] Wyss-Coray T. Mucke L. (2000). Ibuprofen, inflammation and Alzheimer disease. *Nat. Med.* 6 973–974. 10.1038/79661 10973311

[B177] Wyss-Coray T. Mucke L. (2002). Inflammation in neurodegenerative disease–a double-edged sword. *Neuron* 35 419–432. 10.1016/s0896-6273(02)00794-8 12165466

[B178] Wyss-Coray T. Rogers J. (2012). Inflammation in Alzheimer disease-a brief review of the basic science and clinical literature. *Cold Spring Harb. Perspect. Med.* 2:a006346. 10.1101/cshperspect.a006346 22315714 PMC3253025

[B179] Xia M. Q. Qin S. X. Wu L. J. Mackay C. R. Hyman B. T. (1998). Immunohistochemical study of the beta-chemokine receptors CCR3 and CCR5 and their ligands in normal and Alzheimer’s disease brains. *Am. J. Pathol.* 153 31–37. 10.1016/s0002-9440(10)65542-3 9665462 PMC1852933

[B180] Xie X. Ma G. Li X. Zhao J. Zhao Z. Zeng J. (2023). Activation of innate immune cGAS-STING pathway contributes to Alzheimer’s pathogenesis in 5×FAD mice. *Nat. Aging* 3 202–212. 10.1038/s43587-022-00337-2 37118112

[B181] Yan S. D. Chen X. Fu J. Chen M. Zhu H. Roher A. et al. (1996). RAGE and amyloid-beta peptide neurotoxicity in Alzheimer’s disease. *Nature* 382 685–691. 10.1038/382685a0 8751438

[B182] Yang J. Wise L. Fukuchi K.-I. (2020). TLR4 cross-talk with NLRP3 inflammasome and complement signaling pathways in Alzheimer’s disease. *Front. Immunol.* 11:724. 10.3389/fimmu.2020.00724 32391019 PMC7190872

[B183] Ye J. Jiang Z. Chen X. Liu M. Li J. Liu N. (2016). Electron transport chain inhibitors induce microglia activation through enhancing mitochondrial reactive oxygen species production. *Exp. Cell. Res.* 340 315–326. 10.1016/j.yexcr.2015.10.026 26511505

[B184] Zhang N. Liu J. Guo R. Yan L. Yang Y. Shi C. et al. (2024). Palmitoylation licenses RIPK1 kinase activity and cytotoxicity in the TNF pathway. *Mol. Cell.* 84 4419–4435.e10. 10.1016/j.molcel.2024.10.002 39471814

[B185] Zhang X. Shi H. Wu J. Zhang X. Sun L. Chen C. et al. (2013). Cyclic GMP-AMP containing mixed phosphodiester linkages is an endogenous high-affinity ligand for STING. *Mol. Cell.* 51 226–235. 10.1016/j.molcel.2013.05.022 23747010 PMC3808999

[B186] Zhao P. Xu Y. Jiang L. Fan X. Li L. Li X. et al. (2022). A tetravalent TREM2 agonistic antibody reduced amyloid pathology in a mouse model of Alzheimer’s disease. *Sci. Transl. Med.* 14:eabq0095. 10.1126/scitranslmed.abq0095 36070367

[B187] Zhao Y. Wu X. Li X. Jiang L.-L. Gui X. Liu Y. et al. (2018). TREM2 Is a receptor for β-amyloid that mediates microglial function. *Neuron* 97 1023–1031.e7. 10.1016/j.neuron.2018.01.031 29518356 PMC5889092

[B188] Zhou M. Xu R. Kaelber D. C. Gurney M. E. (2020). Tumor Necrosis Factor (TNF) blocking agents are associated with lower risk for Alzheimer’s disease in patients with rheumatoid arthritis and psoriasis. *PLoS One* 15:e0229819. 10.1371/journal.pone.0229819 32203525 PMC7089534

[B189] Zuroff L. Daley D. Black K. L. Koronyo-Hamaoui M. (2017). Clearance of cerebral Aβ in Alzheimer’s disease: Reassessing the role of microglia and monocytes. *Cell. Mol. Life Sci.* 74 2167–2201. 10.1007/s00018-017-2463-7 28197669 PMC5425508

